# Signalling mechanisms mediating Zn^2+^-induced TRPM2 channel activation and cell death in microglial cells

**DOI:** 10.1038/srep45032

**Published:** 2017-03-21

**Authors:** Sharifah Syed Mortadza, Joan A. Sim, Martin Stacey, Lin-Hua Jiang

**Affiliations:** 1 School of Biomedical Sciences, Faculty of Biological Sciences, University of Leeds, United Kingdom; 2 School of Life Sciences, University of Manchester, United Kingdom; 3 School of Molecular and Cell Biology, Faculty of Biological Sciences, University of Leeds, United Kingdom; 4 Sino-UK Joint Laboratory of Brain Function and Injury, and Department of Physiology and Neurobiology, Xinxiang Medical University, PR China

## Abstract

Excessive Zn^2+^ causes brain damage via promoting ROS generation. Here we investigated the role of ROS-sensitive TRPM2 channel in H_2_O_2_/Zn^2+^-induced Ca^2+^ signalling and cell death in microglial cells. H_2_O_2_/Zn^2+^ induced concentration-dependent increases in cytosolic Ca^2+^ concentration ([Ca^2+^]_c_), which was inhibited by PJ34, a PARP inhibitor, and abolished by TRPM2 knockout (TRPM2-KO). Pathological concentrations of H_2_O_2_/Zn^2+^ induced substantial cell death that was inhibited by PJ34 and DPQ, PARP inhibitors, 2-APB, a TRPM2 channel inhibitor, and prevented by TRPM2-KO. Further analysis indicate that Zn^2+^ induced ROS production, PARP-1 stimulation, increase in the [Ca^2+^]_c_ and cell death, all of which were suppressed by chelerythrine, a protein kinase C inhibitor, DPI, a NADPH-dependent oxidase (NOX) inhibitor, GKT137831, a NOX1/4 inhibitor, and Phox-I2, a NOX2 inhibitor. Furthermore, Zn^2+^-induced PARP-1 stimulation, increase in the [Ca^2+^]_c_ and cell death were inhibited by PF431396, a Ca^2+^-sensitive PYK2 inhibitor, and U0126, a MEK/ERK inhibitor. Taken together, our study shows PKC/NOX-mediated ROS generation and PARP-1 activation as an important mechanism in Zn^2+^-induced TRPM2 channel activation and, TRPM2-mediated increase in the [Ca^2+^]_c_ to trigger the PYK2/MEK/ERK signalling pathway as a positive feedback mechanism that amplifies the TRPM2 channel activation. Activation of these TRPM2-depenent signalling mechanisms ultimately drives Zn^2+^-induced Ca^2+^ overloading and cell death.

Microglial cells represent the resident macrophage cells in the central nervous system (CNS). It is widely recognized that microglia cell-mediated inflammatory responses plays an important part in brain injury and neurodegenerative diseases, including hypoxia^1^, ischemic stroke^2^,^3^, multiple sclerosis^4^,^5^,^6^ and Alzheimer’s disease^7^,^8^,^9^,^10^,^11^. Microglial cells can be activated by structurally diverse signals known as damage-associated molecular pattern molecules (DAMPs), including trace metal zinc ion (Zn^2+^)^12^, as well as pathogen-associated molecular pattern molecules^13^. In the brain, Zn^2+^ is mostly concentrated within presynaptic vesicles at the glutamatergic terminal^14^ and released following neuronal stimulation. While Zn^2+^ is crucial for maintaining normal brain functions, excessive Zn^2+^ causes cell death, leading to brain diseases^15^,^16^,^17^ and CNS diseases^12^,^18^.

The signalling mechanisms responsible for Zn^2+^-induced cell death are not fully elucidated. Previous studies suggest that Zn^2+^ can induce cytotoxicity via multiple signalling mechanisms including activation of protein kinase C (PKC)^18^,^19^,^20^, mitochondrial dysfunction^21^,^22^, inhibition of energy production^23^,^24^,^25^ and activation of extracellular signal-regulated kinase (ERK)^26^. Production of reactive oxygen species (ROS) represents the most common component or sequelae of all these signalling mechanisms^12^,^19^,^26^,^27^,^28^. There is increasing evidence to show nicotinamide adenine dinucleotide phosphate (NADPH)-dependent oxidases (NOX) as the main source of ROS generation^29^,^30^. NOX comprise transmembrane catalytic and cytosolic subunits and produce superoxide (O_2_^−^), which is converted into hydrogen peroxide (H_2_O_2_), a signalling molecule implicated in a diversity of pathological conditions^31^,^32^. NOX are widely expressed in the CNS, including microglial cells^33^,^34^,^35^ and their activation is associated with numerous CNS diseases such as ischemic stroke, neurodegenerative disease and retinopathy^36^,^37^,^38^,^39^. Previous studies showed that PKC activation promotes translocation of the cytosolic subunits to the plasma membrane and thereby activation of NOX^40^,^41^,^42^.

Cytosolic Ca^2+^ is a ubiquitous signal in a wide range of cell functions, including cell death. Transient receptor potential melastatin-related 2 (TRPM2) channel plays a crucial role in ROS-induced Ca^2+^ signalling, because of its salient Ca^2+^-permeability and potent activation by ROS in many cell types^43^,^44^,^45^,^46^. Recent studies show that TRPM2-mediated Ca^2+^ signalling is important in DAMP- or ROS-induced cytokine production by monocytes^47^ and macrophage cells^48^, and endothelial hyper-permeability^49^,^50^. However, the best recognized role for the TRPM2 channel is to mediate ROS-induced cell death, which has been revealed in recent studies as critical molecular mechanisms for oxidative stress-related pathologies, including paracetamol-induced liver damage^51^, ischemia-induced kidney injury^52^, reperfusion-associated brain damage^53^ and diabetes^54^.

Among others mechanisms including oxidation of the TRPM2 channel to increase its sensitivity to activation by temperature^55^, the major mechanism by which ROS activates the TRPM2 channel is to promote generation of ADP-ribose (ADPR), the TRPM2 channel specific agonist, via engaging poly(ADPR) polymerases (PARP)^56^, particularly PARP-1 that is critical in the DNA repair mechanism^57^,^58^. Over-activation or prolonged activation of PARP-1 can induce cell death by depleting nicotinamide adenine dinucleotide (NAD) and subsequently ATP^59^,^60^. Several studies show that Zn^2+^ stimulates PARP-1 activation^12^,^61^,^62^,^63^ but it remains elusive how this occurs. An early study suggests that the mitogen-activated protein kinase (MAPK) signalling pathway is important in mediating oxidative stress-induced cell death^64^. There is evidence from a recent study to suggest that ROS can activate PARP-1 via extracellular signal-regulated kinase (ERK)^65^. In oligodendrocyte and differentiated PC12 neuronal cells, an elevation in the [Zn^2+^]_c_ stimulates ERK phosphorylation and activation^26^,^66^ and, depending on the severity of stimulation and cell types, ERK activation promotes cell death or survival^26^,^65^,^67^,^68^,^69^,^70^. In monocytes, TRPM2-mediated Ca^2+^ influx triggers H_2_O_2_-induced MEK/ERK signalling pathway to drive chemokine expression via Ca^2+^-sensitive PYK2 tyrosine kinase^47^.

In the present study, we investigated the role for the TRPM2 channel in Zn^2+^-induced Ca^2+^ signalling and cell death in microglial cells and the mechanisms by which Zn^2+^ activates the TRPM2 channel. Our results show that the TRPM2 channel plays a key role in Zn^2+^-induced increase in the [Ca^2+^]_c_ and cell death. We provide further evidence to indicate that PKC/NOX-mediated generation of ROS and activation of PARP-1 is critical in Zn^2+^-induced TRPM2-mediated Ca^2+^ signalling, which triggers the PYK2/MEK/ERK pathway as a feedback mechanism that amplifies Zn^2+^-induced activation of PARP-1 and TRPM2 channel. Activation of these TRPM2-dependent signalling mechanisms ultimately result in Ca^2+^ overloading leading to microglial cell death.

## Results

### A role of TRPM2 channel in ROS-induced increase in [Ca^2+^]_c_ and cell death in microglial cells

**Figure 1:**
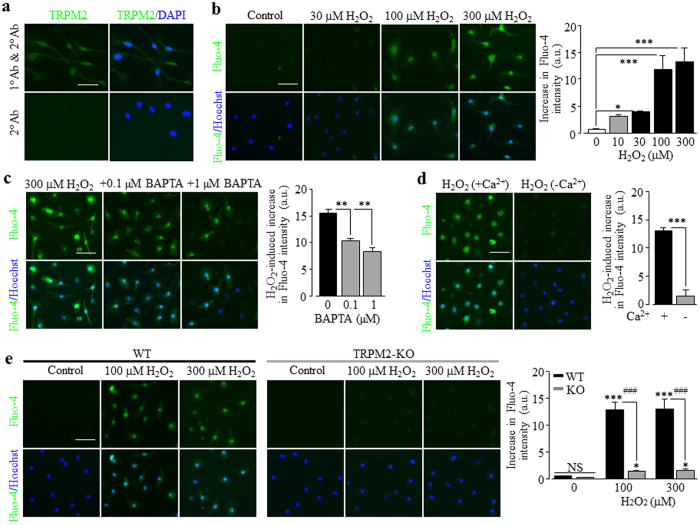
Expression of TRPM2 channel and its role in ROS-induced increase in [Ca^2+^]_c_ in microglial cells. (**a**) Representative images showing TRPM2 immunoreactivity in microglial cells labelled with an anti-TRPM2 antibody. Cells were count-stained with DAPI. Similar results were observed in three independent cell preparations. (**b**–**e**) *Left*, representative single cell images showing Ca^2+^ responses in microglial cells (top row: Fluo-4 fluorescence; bottom row: counter-staining with Hoechst). *Right*, summary of the mean H_2_O_2_-induced Ca^2+^ responses under indicated conditions from four independent experiments, using three wells of cells for each condition in each experiment. The conditions are as follows: microglial cells were exposure to 10–300 μM H_2_O_2_ (**b**), 300 μM H_2_O_2_ without or with treatment with 0.1 and 1 μM BAPTA-AM (**c**), 300 μM H_2_O_2_ in the presence and absence of Ca^2+^ in extracellular solutions (**d**), and 100 and 300 μM H_2_O_2_ in cells from the WT and TRPM2-KO mice (**e**). Cells were treated with BAPTA-AM for 30 min prior to and during exposure to H_2_O_2_. Scale bar, 40 μm. *p < 0.01; ***p < 0.005 compared to indicated control group and, ^###^p < 0.005 compared between the WT and TRPM2-KO cells under the same treatment.

We started with using immunofluorescent confocal microscopy to confirm the TRPM2 expression in microglial cells^71^,^72^,^73^,^74^,^75^. Positive immunostaining was observed in cells labelled with an anti-TRPM2 antibody but not in control cells (Fig. 1a). Exposure to 10–300 μM H_2_O_2_ induced concentration-dependent increases in the [Ca^2+^]_c_ (Fig. 1b). Such Ca^2+^ response was significantly attenuated in cells pre-loaded with 0.1–1 μM BAPTA-AM, a Ca^2+^ chelator (Fig. 1c). H_2_O_2_ evoked negligible increase in the [Ca^2+^]_c_ in extracellular Ca^2+^-free solutions (Fig. 1d), indicating predominant origin from extracellular Ca^2+^ influx. Furthermore, H_2_O_2_-induced increase in the [Ca^2+^]_c_ was significantly inhibited by PJ34 (Supplemental Fig. 1a), a PARP inhibitor known to be critical for oxidative stress-induced TRPM2 channel activation^56^. Finally, exposure to 100–300 μM H_2_O_2_ only induced small increases in the [Ca^2+^]_c_ in the TRPM2-KO microglial cells (Fig. 1e). Taken together, these results provide evidence to support that expression of functional TRMP2 channels plays a key role in mediating ROS-induced Ca^2+^ signalling in microglial cells. While there was no discernible [Ca^2+^]_c_ at 22 °C and 37 °C, reducing temperature from 37 °C to 22 °C significantly attenuated H_2_O_2_-induced increases in the [Ca^2+^]_c_ (Supplemental Fig. 1b,c), indicating that body temperature enhances H_2_O_2_-induced TRPM2 channel activation, as described in pancreatic β-cells^76^. These results are however different from recent studies reporting that body temperature can activate the TRPM2 channel in neurons^77^,^78^.

**Figure 2:**
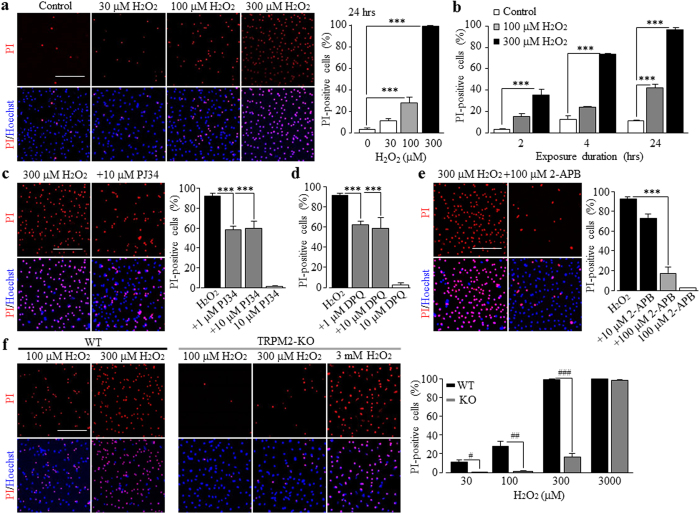
A role for the TRPM2 channel in H_2_O_2_-induced microglial cell death. (**a,c,e,f**) *Left*, representative images showing microglial cell death (top row: PI-stained dead cells; bottom row: all cells counter-stained with Hoechst). *Right*, summary of the mean H_2_O_2_-induced cell death under indicated conditions. The conditions are as follows: cells were exposed for 24 hrs to H_2_O_2_ at indicated concentrations (**a**), 300 μM H_2_O_2_ without or with treatment with 1–10 μM PJ34 (**c**), 1–10 μM DPQ (**d**) or 10–100 μM 2-APB (**e**), H_2_O_2_ at indicated concentrations in the WT and TRPM2-KO cells (f). Cells were treated with PJ34 or 2-APB for 30 min prior to and during exposure to H_2_O_2_. (**b,d**) Summary of the mean cell death induced by exposure to 100 μM and 300 μM H_2_O_2_ for 2, 4 and 24 hrs (**b**), and 300 μM H_2_O_2_ alone and with pre-treatment with 1–10 μM DPQ (**d**) for 30 min. Cells were also treated with each inhibitor at the higher concentration used alone without exposure to H_2_O_2_ (**c–e**). The mean data were from four independent experiments, using three wells of cells for each condition in each experiment. Scale bar, 20 μm. ***p < 0.005 compared to indicated control group and, ^###^p < 0.005 compared between the WT and TRPM2-KO cells under the same treatment.

**Figure 3:**
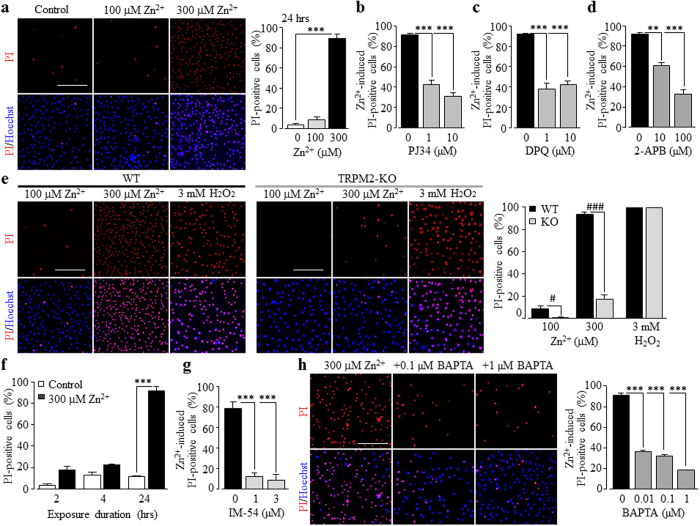
A role of the TRPM2 channel in Zn^2+^-induced microglial cell death. (**a,e,h**) *Left*, representative images showing microglial cell death (top row: PI-stained dead cells; bottom row: all cells counter-stained with Hoechst). *Right*, summary of the mean percentage of cell death induced by Zn^2+^ under indicated conditions. The conditions are as follows: cells were exposed for 24 hrs to Zn^2+^ at indicated concentrations (**a**), Zn^2+^ at indicated concentration in the WT and TRPM2-KO cells (**e**), and 300 μM Zn^2+^ alone or together with BAPTA-AM at indicated concentrations (**h**). Cells were treated with BAPTA-AM for 30 min prior to and during exposure to Zn^2+^. Scale bar, 20 μm. (**b–d,f,g**) Summary of the mean percentage of cell death induced by Zn^2+^ under indicated conditions from at least 3 independent experiments, using three wells of cells for each condition in each experiment. ***p < 0.005 compared to indicated control group and, ^###^p < 0.005, compared between the WT and TRPM2-KO under the same treatment.

**Figure 4:**
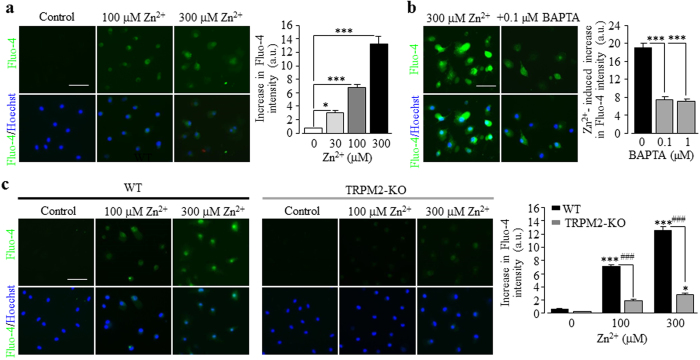
A role of the TRPM2 channel in Zn^2+^-induced increase in the [Ca^2+^]_c_ in microglial cells. (**a–c**) *Left*, representative single cell images showing Ca^2+^ responses in microglial cells (top row: Fluo-4 fluorescence; bottom row: counter-staining with Hoechst). *Right*, summary of the mean Zn^2+^-induced Ca^2+^ responses in microglial cells under indicated conditions from three independent experiments, using three wells of cells for each condition in each experiment. The conditions are as follows: cells were exposed to Zn^2+^ at indicated concentrations (**a**), 300 μM Zn^2+^ in cells without and with treatment with 0.1–1 μM BAPTA-AM (**b**), and Zn^2+^ at indicated concentrations in WT and TRPM2-KO cells (**c**). Cells were treated with BAPTA-AM for 30 min prior to and during exposure to Zn^2+^. Scale bar, 40 μm. *p < 0.01; ***p < 0.005 compared to indicated control group and, ^###^p < 0.005 compared between the WT and TRPM2-KO cells under the same treatment.

**Figure 5:**
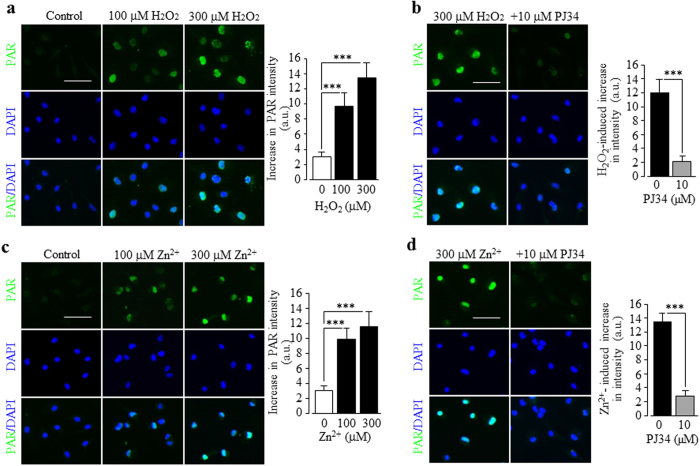
Induction by H_2_O_2_ and Zn^2+^ of PARP-1 activation in microglial cells. (**a**–**d**) *Left*, representative images showing PAR staining (top row) and DAPI (middle row) and merged images (bottom row) of cells without (control) or with exposure for 2 hrs to 100 and 300 μM H_2_O_2_ (**a**), 300 μM H_2_O_2_ alone or together with 10 μM PJ34 (**b**), 100 μM and 300 μM Zn^2+^ (**c**), 300 μM Zn^2+^ alone or together with 10 μM PJ34 (**d**). Cells were treated with PJ34 for 30 min prior to and during exposure to H_2_O_2_ or Zn^2+^. Scale bar, 40 μm. *Right*, summary of the mean PAR fluorescence intensity in cells under indicated conditions from three independent experiments, using three wells of cells for each condition in each experiment. ***p < 0.005 compared to indicated control group.

**Figure 6:**
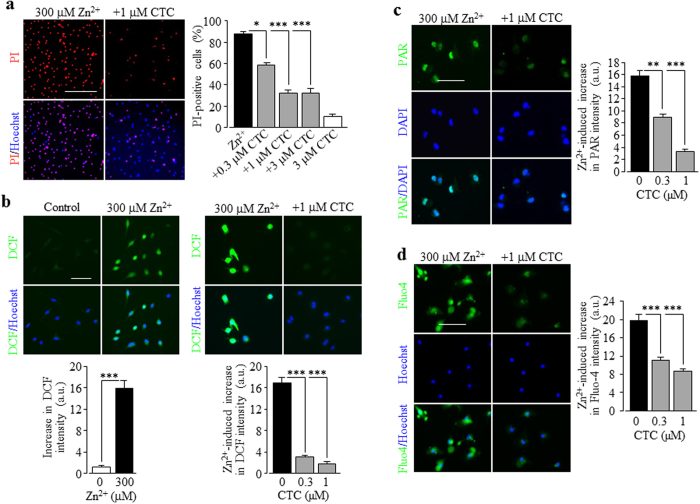
Involvement of PKC activation in Zn^2+^-induced cell death, ROS generation, PARP-1 activation, increase in the [Ca^2+^]_c_ in microglial cells. (**a**) *Left*, representative images showing cell death (top row: PI-stained dead cells; bottom row: all cells counter-stained with Hoechst) in control or chelerythrine chloride (CTC)-treated cells after exposed to 300 μM Zn^2+^ for 24 hrs. *Right,* summary of the mean percentage of cell death from three independent experiments, using three wells of cells for each condition in each experiment. Cells were treated with CTC for 30 min prior to and during exposure to Zn^2+^. (**b**) *Top*, representative images showing ROS level (top row: DCF fluorescence; bottom row: counter-staining with Hoechst) in cells without (control) and with exposure to 300 μM Zn^2+^ for 2 hrs. *Bottom*, summary of the mean Zn^2+^-induced ROS production in microglial cells under indicated conditions from three independent experiments, using three wells of cells for each condition in each experiment. (**c**) *Left*, representative images showing PAR staining (top row) and DAPI (middle row) and merged images (bottom row) of cells exposed for 2 hrs to 300 μM Zn^2+^ alone or together with 1 μM CTC. Cells were treated with CTC for 30 min prior to and during exposure to Zn^2+^. *Right*, summary of the mean PAR fluorescence intensity in cells under indicated concentrations from three independent experiments, using three wells of cells for each condition in each experiment. (**d**) *Left*, representative single cell images showing Ca^2+^ responses in microglial cells (top row: Fluo-4 fluorescence; bottom row: counter-staining with Hoechst). *Right*, summary of the mean Zn^2+^-induced Ca^2+^ responses in microglial cells under indicated conditions from three independent experiments, using three wells of cells for each condition in each experiment. Scale bar, 20 μm (**a**) and 40 μm (**b–d**). *p < 0.05; ***p < 0.005 compared to indicated control group exposed to with Zn^2+^ alone. Treatment with the highest concentration of CTC (**a**) alone resulted in no significant cell death.

**Figure 7:**
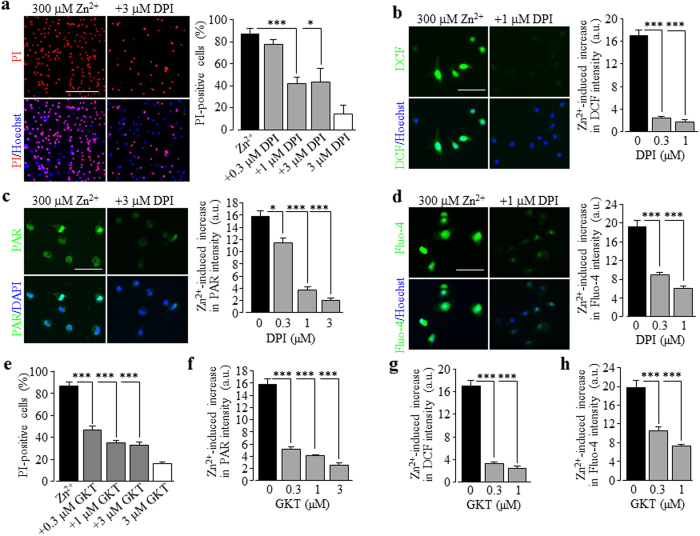
Involvement of NADPH oxidase activation in Zn^2+^-induced cell death, ROS generation, PARP-1 activation, increase in the [Ca^2+^]_c_ in microglial cells. (**a**) *Left* representative images showing cell death (top row: PI-stained dead cells; bottom row: all cells counter-stained with Hoechst) in microglial cells exposed for 24 hrs to 300 μM Zn^2+^ alone or together with 3 μM DPI. (**b**) *Left*, representative images showing DCF fluorescence (top row) and counter-staining with Hoechst (bottom row) in cells exposed for 2 hrs to 300 μM Zn^2+^ alone or together with 1 μM DPI (**b**). (**c**) *Left* representative images showing PAR staining (top row) and counter-staining with DAPI (bottom row) of cells exposed for 2 hrs to 300 μM Zn^2+^ alone or together with 3 μM DPI. (**d**) *Left*, representative single cell images showing Ca^2+^ responses in microglial cells (top row: Fluo-4 fluorescence; bottom row: counter-staining with Hoechst) exposed for 2 hrs to 300 μM Zn^2+^ alone or together with for 2 hrs to 300 μM Zn^2+^ alone or together with 1 μM DPI (d). Cells were treated with DPI or GKT for 30 min prior to and during exposure to Zn^2+^. Scale bar, 20 μm (**a**) and 40 μm (all other panels). (**a–h**) Summary of the mean data from three independent experiments, using three wells of cells for each condition in each experiment. *p < 0.05; ***p < 0.005 compared to the indicated control group exposed to with Zn^2+^ alone. Treatment with the highest concentration of DPI (**a**) or GKT (**e**) alone resulted in no significant cell death.

**Figure 8:**
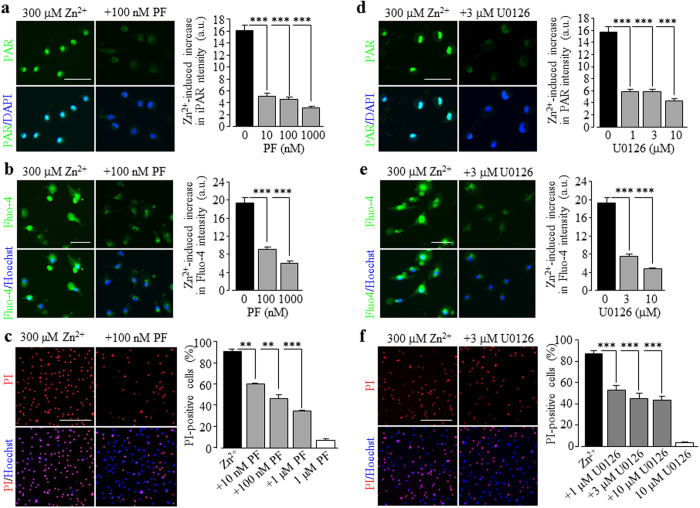
Engagement of the PYK2/MEK/ERK signalling pathway in Zn^2+^-induced PARP-1 activation increase in the [Ca^2+^]_c_ and microglial cell death. (**a**,**d**) *Left*, representative images showing the PAR level (top row: PAR fluorescence; bottom row: counter-staining with DAPI) in microglia cells exposed for 2 hrs to 300 μM Zn^2+^ alone or together with 100 nM PF 431396 (PF) (**a**) or 3 μM U0126 (**d**). *Right*, summary of the mean PAR fluorescence intensity in cells under indicated conditions from three independent experiments, using three wells of cells for each condition in each experiment. (**b,e**) *Left*, representative single cell images showing Ca^2+^ responses (top row: Fluo-4 fluorescence; bottom row: counter-staining with Hoechst) in microglial cells to 300 μM Zn^2+^ without and with treatment with 100 nM PF (**b**) or 3 μM U0126 (**e**). *Right*, summary of the mean Zn^2+^-induced Ca^2+^ responses in microglial cells under indicated conditions from three independent experiments, using three wells of cells for each condition in each experiment. (**c,f**) *Left*, representative images showing cell death (top row: PI-stained dead cells; bottom row: all cells counter-stained with Hoechst) in microglial cells exposed for 24 hrs to 300 μM Zn^2+^ in cells without and with treatment with 100 nM PF (**c**) or 3 μM U0126 (**f**). *Right*, summary of the mean percentage of cell death from three independent experiments, using three wells of cells for each condition in each experiment. Cells were treated with PF or U0126 for 30 min prior to and during exposure to Zn^2+^. Scale bar, 40 μm (**a,b,d,e**) and 20 μm (**c, f**). *p < 0.05; ***p < 0.005 compared to the indicated control group exposed to with Zn^2+^ alone. Treatment with the highest concentration of PF (**c**) or U0126 (**f**) alone resulted in no significant cell death.

**Figure 9:**
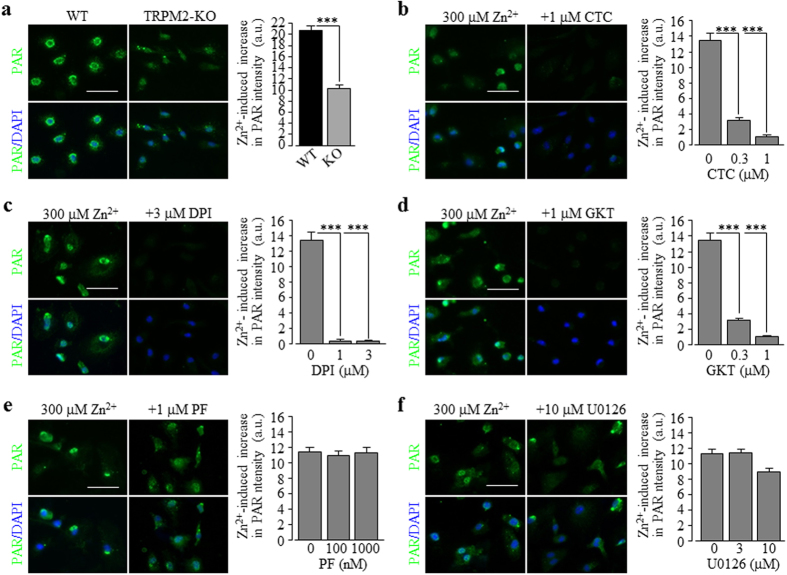
The PKC/NOX signalling pathway is required for, and the PYK2/MEK signalling pathway depends on, the TRPM2 channel activation. (**a**) *Left*, representative images showing the PAR level (top row: PAR fluorescence; bottom row: counter-staining with DAPI) in the WT and TRPM2-KO microglia cells exposed for 2 hrs to 300 μM Zn^2+^. *Right*, summary of the mean Zn^2+^-induced PAR fluorescence intensity in the WT and TRPM2-KO cells from three independent experiments, using three wells of cells for each condition in each experiment. (**b–f**) *Left*, representative images showing the PAR level (top row: PAR fluorescence; bottom row: counter-staining with DAPI) in the TRPM2-KO microglia cells exposed for 2 hrs to 300 μM Zn^2+^ alone or together with 1 μM CTC (**b**), 3 μM DPI (**c**), 1 μM GKT (**d**), 1 μM PF 431396 (PF) (**e**) or 10 μM U0126 (f). *Right*, summary of the mean PAR fluorescence intensity in microglial cells under indicated conditions from at least three independent experiments, using three wells of cells for each condition in each experiment. Scale bar, 40 μm. ***p < 0.005 compared to the WT cells (**a**) or cells exposed to Zn^2+^ alone (**b–f**). The Zn^2+^-induced residual PAR generation in the TRPM2-KO microglial cells was strongly inhibited or abolished by treatment with CTC (**b**), DPI (**c**) or GKT (**d**), but not with PF (**e**) or U0126 (**f**). Scale bar, 40 μm. ***p < 0.005 compared to indicated control group treated with PF or U0126 alone, and ^###^p < 0.005 compared to cells exposed to Zn^2+^ and treated with PF or U0126.

**Figure 10:**
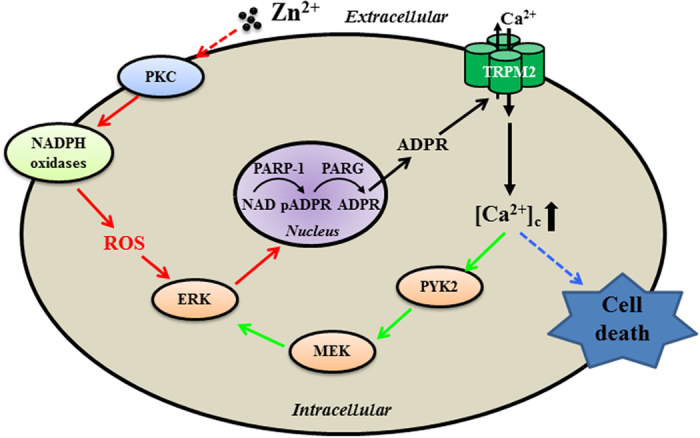
Schematic summary of the signalling mechanisms mediating Zn^2+^-induced TRPM2 channel activation and cell death in microglial cells. TRPM2 channels are expressed in microglial cells as Ca^2+^-permeable cationic channel on the cell surface. Zn^2+^ activates the TRPM2 channel involving multiple-step intracellular signalling pathways in microglia cell death. Zn^2+^ stimulates PKC and NADPH oxidases, ROS generating-enzyme. ROS activates PARP-1 and PARG in the nucleus leading to ADPR production and subsequent activation of TRPM2-dependent Ca^2+^ influx to increase the cytoplasmic Ca^2+^ concentrations ([Ca^2+^]_c_). Elevated [Ca^2+^]_c_ in turn activate the PYK2/MEK/ERK signalling pathway as a positive feedback mechanism that amplifies activation of PARP-1, leading to TRPM2-mediated Ca^2+^ overloading and cell death. Abbreviations: PKC, protein kinase C; NADPH oxidase, nicotinamide adenine dinucleotide phosphate-dependent oxidase; ROS, reactive oxygen species; ERK, extracellular signal-regulated kinase; NAD, nicotinamide adenine dinucleotide; pADPR, poly(ADP-ribose) moiety; ADPR, ADP-ribose; PARP-1, poly(ADP-ribose) polymerase 1; PARG, poly(ADP-ribose) glycohydrolase; MEK, mitogen-activated kinase; PYK2, protein tyrosine kinase 2.

As introduced above, the common role for the TRPM2 channel established in diverse cell types is to mediate ROS-induced cell death^56^,^79^,^80^,^81^. However, it was yet unclear whether the TRPM2 channel has such a role in ROS-induced microglial cell death, but such information is important for a better understanding of microglial cells in oxidative stress-related pathologies. We therefore examined H_2_O_2_-induced microglial cell death, using PI staining. Exposure to 30–300 μM H_2_O_2_ for 24 hrs evoked concentration-dependent increases in cell death (Fig. 2a). H_2_O_2_-induced cell death was also dependent of the exposure duration, increasing markedly as the exposure duration was extended from 2 hrs to 4 and 24 hrs (Fig. 2b). H_2_O_2_-induced cell death was attenuated by IM-54, a necrosis inhibitor, but insensitive to Ac-DEVD-CMK, an inhibitor of caspase-dependent apoptosis (Supplemental Fig. 2a,b). H_2_O_2_-induced cell death was considerably suppressed by 1–10 μM PJ34 (Fig. 2c) or 1–10 μM DPQ (Fig. 2d), two structurally different PARP inhibitors, and also strongly inhibited by 100 μM 2-APB (Fig. 2e), an inhibitor known to block the TRPM2 channel, while treatment with any of these inhibitors alone resulted in minimal cell death (Fig. 2c–e). Moreover, H_2_O_2_-induced cell death was attenuated by BAPTA-AM at 1 μM, but not at lower concentrations (10–100 nM) (Supplemental Fig. 2c). In striking contrast with what observed in the WT microglial cells, exposure to 30–300 μM H_2_O_2_ for 24 hrs caused no or modest cell death in the TRPM2-KO microglial cells (Fig. 2f). As a positive control, exposure to 3 mM H_2_O_2_ in parallel experiments caused massive cell death that was not different between the WT and TRPM2-KO microglial cells (Fig. 2f). These results therefore provide compelling evidence to support a role for the TRPM2 channel in mediating ROS-induced Ca^2+^ signalling and cell death in microglial cells.

### A role of TRPM2 channel in Zn^2+^-induced increase in [Ca^2+^]_c_ and cell death in microglial cells

As introduced above, excessive Zn^2+^ is cytotoxic. Exposure of microglial cells to 100–300 μM Zn^2+^ for 24 hrs resulted in concentration-dependent cell death (Fig. 3a). Zn^2+^ can promote ROS production^12^,^26^,^28^, promoting us to examine whether the TRPM2 channel plays a role in mediating Zn^2+^-induced microglial cell death. Zn^2+^-induced cell death was significantly reduced by 1–10 μM PJ34, 1–10 μM DPQ or 10–100 μM 2-APB (Fig. 3b–d and Supplemental Fig. 3). Furthermore, cell death induced by 100–300 μM Zn^2+^ was largely abolished in the TRPM2-KO microglial cells (Fig. 3e). These results clearly demonstrate that the TRPM2 channel activity as well as the PARP activity is critical in Zn^2+^-induced microglial cell death. Of notice, Zn^2+^-induced cell death exhibited strong dependence of exposure duration and occurred at a significant level only after the duration was prolonged to 24 hrs (Fig. 3f). Zn^2+^-induced cell death, while remaining insensitive to 10–30 μM Ac-DEVD-CMK, was almost completely inhibited by 1–3 μM IM-54 (Fig. 3g; Supplemental Fig. 4). Furthermore, Zn^2+^-induced cell death was strongly reduced in cells pre-loaded with BAPTA-AM even at 10–100 nM as well as at 1 μM (Fig. 3h), suggesting that an increase in the [Ca^2+^]_c_ is critical in Zn^2+^-induced cell death.

### Involvement of Zn^2+^-induced stimulation of PARP-1 activity in TRPM2 channel activation

To further demonstrate that exposure to Zn^2+^ causes cell death via TRPM2 channel activation, we returned to single cell imaging to monitor Zn^2+^-induced change in the [Ca^2+^]_c_ in microglial cells. Application of 30–300 μM Zn^2+^ for 2 hrs, although evoking no significant cell death (Fig. 3f), gave rise to strong and concentration-dependent increase in the [Ca^2+^]_c_ (Fig. 4a). Such Ca^2+^ response was significantly attenuated in cells preloaded with 0.1–1 μM BAPTA-AM (Fig. 4b). Like H_2_O_2_-induced increase in the [Ca^2+^]_c_, Zn^2+^-induced increase in the [Ca^2+^]_c_ was also reduced by decreasing temperature from 37 °C to 22 °C (Supplemental Fig. 5a,b). Furthermore, Zn^2+^-induced increase in the [Ca^2+^]_c_ was suppressed by PJ34 (Supplemental Fig. 5c) and almost lost in the TRPM2-KO microglial cells (Fig. 4c). Taken together, these results strongly support that exposure to Zn^2+^ induces TRPM2 channel activation. An important question arising from such a finding is that how the TRPM2 channel is activated in response to exposure to Zn^2+^. An increase in the PARP-1 activity in the nucleus represents a major mechanism by which oxidative stress induces the TRPM2 channel activation^56^. To provide direct evidence to show whether the PARP-1 activity is critical, we performed immunostaining using an antibody that recognizes PAR, the product of PARP activity. As a positive control, exposure to 100–300 μM H_2_O_2_ for 2 hrs stimulated substantial PAR production that was highly concentrated in the nucleus, as evidenced by the co-localization with DAPI nuclear staining (Fig. 5a). These results indicate that H_2_O_2_ mainly stimulates the PARP-1 activity. H_2_O_2_-induced PAR production in the nucleus, as anticipated, was almost completely inhibited by PJ34 (Fig. 5b). Similarly, exposure to 100–300 μM Zn^2+^ for 2 hrs potently promoted PAR generation in the nucleus (Fig. 5c), which was also strongly suppressed by 10 μM PJ34 (Fig. 5d). These results collectively provide strong evidence to support that exposure to Zn^2+^ stimulates the PARP-1 activity and thereby activates the TRPM2 channels in microglial cells.

### A role of PKC and NOX in Zn^2+^ stimulation of ROS production and PARP-1 activity

We were interested in the upstream signalling mechanisms, particularly those generating ROS, which mediate Zn^2+^ stimulation of the PARP-1 activity and TRPM2 channel activation. Previous studies showed that PKC and NOX are crucial in Zn^2+^-induced ROS generation^18^,^19^,^20^. We moved on to examine firstly whether activation of PKC and NOX is involved in Zn^2+^-induced cell death. Treatment with 0.3–3 μM chelerythrine chloride (CTC), a potent PKC inhibitor, strongly and concentration-dependently inhibited Zn^2+^-induced cell death (Fig. 6a). Next, we performed single cell imaging to determine whether exposure to Zn^2+^ promoted ROS production in microglial cells, using DCF, a fluorescent indicator for ROS generation. Exposure to 300 μM Zn^2+^ resulted in a massive increase in the cytosolic ROS level, which was strongly inhibited by 0.3–1 μM CTC (Fig. 6b). Furthermore, treatment with 0.3–1 μM CTC strongly and concentration-dependently inhibited Zn^2+^-induced PAR generation in the nucleus (Fig. 6c) and increase in the [Ca^2+^]_c_ (Fig. 6d). Similarly, Zn^2+^-induced cell death, particularly ROS production, PARP-1 activation and increase in the [Ca^2+^]_c_. was strongly concentration-dependently inhibited by treatment with 0.3–3 μM DPI, a generic NOX inhibitor (Fig. 7a–d), and 0.3–3 μM GKT137831, a NOX1/4 selective inhibitor (Fig. 7e–h; supplemental Fig. 6), and also, albeit to less extent by treatment with 10–30 μM Phox-I2, a NOX2 selective inhibitor (supplemental Fig. 7). Taken together, these results provide clear evidence to show a significant role for PKC and NOX, particularly NOX1/4, in Zn^2+^-induced ROS production and PARP-1 activation, leading to TRPM2 channel activation, increase in the [Ca^2+^]_c_ and cell death in microglial cells.

### The PYK2/MEK/ERK signalling pathway as a feedback mechanism stimulating PARP-1 activity, TRPM2 channel activation, and cell death

Zn^2+^-induced ROS production, stimulation of PARP-1 and increase in the [Ca^2+^]_c_ in microglial cell were observed after exposure to Zn^2+^ for 2 hrs (Figs 4, 5, 6), but Zn^2+^-induced cell death occurred 24 hrs, but not 2–4 hrs after exposure (Fig. 3f), suggesting possible involvement of additional signalling pathways as positive feedback mechanisms. There is evidence to suggest a role of the MEK/ERK signalling in ROS-induced PARP-1 activation^70^. It is also known that the protein tyrosine kinase PYK2 is highly expressed in the central nervous system, including in microglial cells^82^,^83^ and, more importantly, PYK2 is sensitive to activation by Ca^2+^ on one hand and can trigger the MEK/ERK signalling pathway on the other hand^84^,^85^, and thus it is well placed to mediate Ca^2+^-induced activation of the MEK/ERK signalling pathway. Furthermore, TRPM2-mediated Ca^2+^ influx or increase in the [Ca^2+^]_c_ activates the PYK2/MEK/ERK signalling pathway in monocytes^47^. These led us to hypothesize that the initial increase in the [Ca^2+^]_c_, resulting from Zn^2+^-induced TRPM2 activation via the PKC/NOX signalling mechanism, subsequently activates the PYK2/MEK/ERK signalling pathway and further stimulates the PARP-1 activity. To provide evidence to support or refute this hypothesis, we examined the effects of PF431396, a potent PYK2 inhibitor, and U0126, an inhibitor of MEK that phosphorylates and thereby activates the ERK, on Zn^2+^-induced stimulation of PARP-1, increase in the [Ca^2+^]_c_ and cell death. Treatment with 10–1000 nM PF431396 concentration-dependently inhibited but did not completely prevent Zn^2+^-induced PAR production in the nucleus (Fig. 8a), increase in the [Ca^2+^]_c_ (Fig. 8b) and cell death (Fig. 8c). Similarly, treatment with 1–10 μM U0126 caused strong but incomplete inhibition of Zn^2+^-induced stimulation of PARP-1 (Fig. 8d), increase in the [Ca^2+^]_c_ (Fig. 8e) and cell death (Fig. 8f). These results are consistent with the concept that PYK2/MEK/ERK as the signalling mechanism downstream of the TRPM2 channel activation plays an important part in Zn^2+^-induced cell death.

To seek further evidence to support the hypothesis that the PKC/NOX ROS-generating signalling pathway acts as the trigger for the TRPM2 channel activation and the PYK2/MEK/ERK signalling pathway serves as a mechanism downstream of TRPM2 channel activation that promote further TRPM2 channel activation, we attempted further experiments. TRPM2-KO reduced the PAR production in microglial cells (Fig. 9a), and we firstly examined the effects of inhibiting the PKC/NOX signalling pathway with CTC, DPI and GKT137831, and the PYK2/MEK signalling pathway with PF431396 and U0126 on the PARP-1 dependent PAR production in the TRPM2-KO microglial cells. Treatment with 0.3–1 μM CTC (Fig. 9b), 1–3 μM DPI (Fig. 9c) or 0.3–1 μM GKT137831 (Fig. 9d) almost completely abolished Zn^2+^-induced PAR production. In striking contrast, treatment with 100–1000 nM PF431396 (Fig. 9e) or 3–10 μM U0126 (Fig. 9f) resulted in no significant inhibition. Next, we investigated whether Zn^2+^ induced any significant increase in the [Ca^2+^]_c_ in cells pre-treated with 1000 nM PF431396 (Supplemental Fig. 8a) or 10 μM U0126 (Supplemental Fig. 8b) to inhibit the PYK2/MEK signalling pathway. As anticipated, in microglial cells with the PYK2/MEK signalling pathway being inhibited, Zn^2+^ was still able to induce considerable increase in the [Ca^2+^]_c_ and such Zn^2+^-induced increase in the [Ca^2+^]_c_ was abolished by treatment with 1 μM CTC, 3 μM DPI or 1 μM GKT137831 (Supplemental Fig. 9). Taken together, these results provide further supporting evidence to show that the PKC/NOX signalling pathway is required for Zn^2+^-induced PARP-1 activation and thereby TRPM2 channel activation, and the PYK2/MEK/ERK signalling pathway is activated downstream of the TRPM2 channel activation (Fig. 10).

## Discussion

The present study provides pharmacological and genetic evidence to show that the TRPM2 channel acts as a key mechanism mediating Ca^2+^ signalling and cell death in microglial cells in response to exposure to ROS and Zn^2+^ at concentrations reported to be presented in the brain under the pathological conditions. We have revealed Zn^2+^-induced activation of the PKC/NOX signalling mechanism promotes ROS production, PARP-1 activity and TRPM2 channel activation. Furthermore, the PYK2/MEK/ERK signalling pathway acts downstream of the TRPM2 channel activation or TRPM2-mediated increase in the [Ca^2+^]_c_ as a positive feedback mechanism that drives Ca^2+^ overloading and cell death, as illustrated in Fig. 10.

As introduced above, studies over the past years have shown that the Ca^2+^-permeable TRPM2 channel on the cell surface acts as a major molecular mechanism for ROS-induced Ca^2+^ signalling in immune cells^80^. An early study reported that exposure of microglial cells to H_2_O_2_ induced an increase in the [Ca^2+^]_c_^73^ but it was not clearly understood how important the TRPM2 channel was in mediating ROS-induced Ca^2+^ signalling. In the present study, we showed that H_2_O_2_-induced increase in the [Ca^2+^]_c_ in microglial cells (Fig. 1b–c) was largely abolished in the absence of extracellular Ca^2+^ (Fig. 1d) or lost in the TRPM2-KO microglial cells (Fig. 1f). These results provide compelling evidence to indicate that the cell surface TRPM2 channel plays a major role in ROS-induced Ca^2+^ signalling via mediating Ca^2+^ influx, as previously reported in macrophage cells^45^. This study further showed that exposure to H_2_O_2_ caused considerable cell death in the WT microglial cells (Fig. 2a,b), which was attenuated by PJ34, DPQ or 2-APB (Fig. 2c–e) and, in addition, abolished in the TRPM2-KO microglial cells (Fig. 2f). These results indicate a crucial role for the TRPM2 channel activation in mediating ROS-induced microglial cell death. It is well-known that excessive Zn^2+^ is highly cytotoxic and plays a critical role in mediating neuronal death^21^,^79^,^86^. In the present study, we showed that Zn^2+^ at concentrations observed in the brain under pathological conditions such as ischemia-reperfusion brain damage and epilepsy^87^,^88^, evoked substantial microglial cell death (Fig. 3a). Such microglial cell death was inhibited by PJ34, DPQ and 2-APB (Fig. 3b–d), and particularly absent in the TRPM2-KO microglial cells (Fig. 3e), supporting a critical role for the TRPM2 channel activation. Exposure to Zn^2+^ elicited considerable increase in the [Ca^2+^]_c_ (Fig. 4a), which was strongly reduced by PJ34 (Supplemental Fig. 1b) and largely absent in the TRPM2-KO microglial cells (Fig. 4c). Buffering TRPM2-mediated increase in the [Ca^2+^]_c_ with 10–100 nM BAPTA-AM (Fig. 4b) strongly attenuated Zn^2+^-induced cell death (Fig. 3h), suggesting the importance of TRPM2-mediated Ca^2+^ influx or Ca^2+^ signalling in Zn^2+^-induced cell death. As far as we are aware, the present study is the first to reveal TRPM2 channel activation, particularly TRPM2-mediated increase in the [Ca^2+^]_c_, as a mechanism contributing to Zn^2+^ cytotoxicity. H_2_O_2_-mediated TRPM2-mediated microglial cell death was significantly inhibited (Fig. 1f; Supplemental Fig. 2a) and particularly Zn^2+^-induced TRPM2-mediated microglial cell death (Fig. 3e; Supplemental Fig. 3a) was almost completely prevented by the necrosis inhibitor IM-54, suggesting necrotic cell death, a mechanism eliciting inflammatory responses. This is consistent with the findings from recent transgenic studies that the TRPM2 channel in microglial cells is engaged in inflammatory pain^71^ and post-ischemic stroke brain damage^75^ and AD^80^,^89^.

We further investigated the signalling mechanisms by which exposure to Zn^2+^ induces TRPM2 channel activation in microglial cells. In microglial cells, Zn^2+^ is known as a potent inhibitor for voltage-gated proton channel which functions to promote NOX-dependent ROS production in microglia^90^. The present study revealed that like H_2_O_2_, Zn^2+^ stimulated PARP-1 dependent PAR generation in the nucleus (Fig. 5). It is known that NOX represents an important source for ROS generation in the brain and PKC stimulates NOX. Consistently, Zn^2+^-induced ROS production, PARP-1 activity, and cell death were strongly reduced by inhibiting PKC (Fig. 6) and NOX (Fig. 7a–d), including NOX1/4 (Fig. 7e–h) and NOX2 (Supplemental Fig. 7). These results provide strong evidence to show that PKC/NOX-mediated ROS generation is critical in Zn^2+^-induced stimulation of PARP-1 activity, TRPM2-mediated increase in the [Ca^2+^]_c_ and cell death in microglial cells. Previous studies suggest that ROS can stimulate PARP-1 via the MEK/ERK signalling. In monocytes, TRPM2-mediated Ca^2+^ influx activates the PYK2/MEK/ERK signalling pathway in response to H_2_O_2_ *in vitro* or oxidative stress *in vivo*, which is important in chemokine generation^47^. Here, we show that Zn^2+^-induced stimulation of PARP-1, increase in the [Ca^2+^]_c_ and cell death was strongly suppressed by inhibiting the PYK2/MEK/ERK signalling pathway (Fig. 8d,e). It is worth mentioning the inhibitors used in the study are limited in their specificity, and nonetheless, our results are consistent with the hypothesis that the PYK2/MEK/ERK signalling pathway constitutes a positive feedback mechanism that amplifies Zn^2+^-induced stimulation of PARP-1, TRPM2 channel activation, and increase in the [Ca^2+^]_c_ that ultimately drives cell death. Activation of such signalling mechanisms offers a feasible explanation for the significant delay in Zn^2+^-induced cell death (Fig. 3f). Of notice, exposure to H_2_O_2_ induced stimulation of the PARP-1 activity (Fig. 5a,b), TRPM2-mediated increase in the [Ca^2+^]_c_ (Fig. 1f) and cell death (Fig. 2c–f). However, in striking contrast with Zn^2+^, H_2_O_2_-induced effects were completely insensitive to inhibitors of the PKC/NOX (Supplemental Figs 9 and 10) and PYK2/MEK/ERK signalling mechanisms (Supplemental Fig. 11). These results indicate that H_2_O_2_ induced microglial cell death via stimulating the PARP-1 activity and subsequently TRPM2 channel activation, independent of the PKC/NOX signalling pathway and the PYK2/MEK/ERK signalling pathway. It is worth mentioning that when heterologously expressed in HEK293 cells, the TRPM2 channel in the open state but not in the closed state becomes inactivated upon exposure to extracellular Zn^2+^ at concentrations used in this study^91^. The exact reason for discrepancy in terms of Zn^2+^ inhibition of the endogenously and heterologously overexpressed TRPM2 channels is currently unclear, and may arise from the different TRPM2 expression level. Alternatively or additionally, Zn^2+^ has been rapidly transported by yet defined Zn^2+^-transporting mechanisms into the cytosol in order to induce PKC activation and as a result, extracellular Zn^2+^ concentrations insufficiently inhibit the TRPM2 channel.

In conclusion, the study provides evidence to show TRPM2 channel activation as a critical mechanism mediating ROS/Zn^2+^-induced Ca^2+^ signalling and cell death in microglial cells. We have also revealed that activation of the PKC/NOX signalling pathway is an important mechanism in Zn^2+^-induced stimulation of PARP-1, TRPM2 channel activation, and increase in the [Ca^2+^]_c_ and, additionally, activation of the PYK2/MEK/ERK signalling pathway acts as a positive feedback signalling mechanism that further amplifies stimulation of PARP-1 and TRPM2 channel activation. Activation of these signalling mechanisms in microglial cells, in response to prolonged exposure to excessive Zn^2+^, ultimately drives Ca^2+^ overloading and cell death. The findings reported in this study, despite their relevance to Zn^2+^-related brain damage *in vivo* remaining to be further explored, should help to evolve a better and mechanistic insight into Zn^2+^-induced cytotoxicity.

## Methods

### Chemicals

All chemicals or reagents were obtained from Sigma-Aldrich unless specified otherwise. PJ34 was from Santa Cruz, DPQ from Calbiochem, Ac-DVED-CMK, GKT137831, BAPTA-AM and U0126 from Cayman Chemical, CTC and PF431396 from Tocris.

### Primary microglial cell cultures

All experiments and experimental protocols, including all those involving mice, were approved by the University of Leeds Ethical Review Committee and performed in accordance with the University of Leeds guidelines and procedure and conforming to the UK Home Office rules and regulations. Primary microglia cultures were prepared from 1–3 day old mice. The generation of TRPM2-KO mice was detailed in our previous study^45^. After the mice were sacrificed, the cerebral hemispheres were isolated and, after the meninges were removed under a dissecting microscope, were minced into small pieces. This was followed by incubating the tissues in 0.05% trypsin-EDTA solution for 20 min at 37 °C. The tissues were further dissociated by triturating using a pipette and subsequently filtered using a 70-μm cell strainer. Cells were collected by centrifugation at 1300 rpm for 5 min, and the pellet was re-suspended in 2 ml of DMEM containing high glucose supplemented with 10% FBS, 10 units/ml penicillin, and 100 μg/ml streptomycin. The cell suspension from 2 brains was added to a 75-cm^2^ flask that was pre-coated with poly-L-lysine in total 15 ml of the same culture media. Cells were maintained at 37 °C in a humidified atmosphere of 5% CO_2_. Half of the culture media was replaced with fresh media following 4 day incubation. Cells were continued to be incubated further for 5–8 days. Loosely attached microglial cells were separated from the rest of cell culture by shaking the flasks in a rotary platform in a tissue culture incubator at 37 °C at 180 rpm for 90 min. Microglial cells were collected by centrifuging at 280 *g* for 5 min, re-suspended in fresh culture medium and were seeded in 96-wells plates (Costar) at a density of 1.1 × 10^5^ and 2.75 × 10^5^ cells/ml for cell death assays and Ca^2+^ imaging, respectively. For immunostaining, cells were seeded onto poly-L-lysine coated coverslips at 5 × 10^4^ cells/ml and placed in 24-well plates (Costar). Cells were incubated for another 72 hrs before used for experiments.

### Single cell Ca^2+^ imaging

This was performed on live cells, as described above, which were seeded in 96-well plates and incubated for 72 hrs. After the culture media were removed, cells were washed twice with standard bath solution (SBS in mM: 134 NaCl, 5 KCl, 0.6 MgCl_2_, 1.5 CaCl_2_, 8 glucose and 10 HEPES, pH 7.4) before they were loaded with 5 μg/ml Fluo-4/AM and 0.2% pluronic acid F-127 (Life Technologies) in SBS at 37 °C for 45 min, followed by extensive washing with SBS and maintaining in 200 μl of SBS or Ca^2+^ free-SBS for 30 min at room temperature prior to the application of H_2_O_2_ or Zn^2+^ at indicated concentration at 37° for a majority of experiments and at 22 °C for a small number of experiments as indicated. In experiments using inhibitor, cells were pretreated with inhibitors including BAPTA-AM at indicated concentrations for 30 min at 37 °C prior to the application of 300 μM H_2_O_2_ or 300 μM Zn^2+^. At the end of treatment with H_2_O_2_ or Zn^2+^, cells were counterstained by Hoechst at a concentration of 5 μg/ml. The fluorescent images were captured using an Olympus IX51 microscope, a digital camera and Cell^^F^ software (Olympus). Data analysis was carried out using ImageJ and at least 75 cells were examined in each well.

### Cell death assay

Cell death was assessed by propidium iodide (PI) staining. Cells plated as described above in 96-wells plates were treated with H_2_O_2_ or Zn^2+^ at indicated concentrations. In experiments studying inhibitors, cells were pretreated with indicated inhibitors for 30 min at 37 °C prior to the application of 300 μM H_2_O_2_ or 300 μM Zn^2+^. At the end of treatment with H_2_O_2_ or Zn^2+^, cells were co-stained by PI and Hoechst with a concentration of 2 μg/ml and 5 μg/ml, respectively. The phase contrast and fluorescent images of the cells were captured using an Olympus IX51 microscope, a digital camera and Cell^F^ software (Olympus). The number of PI-stained dead cells and the total number of cells identified by Hoechst-staining in three randomly chosen areas in each image were counted using ImageJ, and at least 100 cells were examined in each well. Cell death was presented by expressing PI-stained cells as percentage of Hoechst-stained cells.

### Immunofluorescent confocal imaging

Cells were fixed with 4% paraformaldehyde (PFA) dissolved in deionized water for 15 min and permeabilized in PBS containing 0.1% Triton X-100. Following rinsing twice with phosphate buffer saline (PBS) containing 0.5% Tween 20 (PBST), cells were blocked in PBS containing 5% goat serum for 30 min. Cells were incubated with the primary rabbit anti-TRPM2 antibody (Bethyl) at a dilution of 1:1500 or mouse anti-PAR antibody (Enzo;1: 500) overnight at room temperature and, after extensive washing in PSBT, incubated with the secondary FITC-conjugated goat anti-rabbit IgG antibody (Sigma; 1:1000) or anti-mouse IgG antibody (Sigma; 1: 1000) for 1 hr at room temperature. After washing with PBS and rinsing in water, coverslips were mounted using florescent mounting medium with 4′,6-diamidino-2-phenylindole (DAPI). All images were captures using an Olympus IX51 microscope, a digital camera and Cell^F^ software (Olympus). The intensity of the fluorescent was quantified using ImageJ and at least 75 cells were examined in each well.

### Measurement of ROS production

The level of reactive oxygen species in cells were measured using florescent probe 2′,7′-dichlorodihydrofluorescein diacetate (DCFH-DA). Briefly, cells were plated in 96 well plates for 72 hrs before use. Cells were treated with H_2_O_2_ or Zn^2+^ at indicated concentrations for 2 hrs. In experiments studying inhibitors, cells were pretreated with indicated inhibitors for 30 min at 37 °C prior to the application of 300 μM H_2_O_2_ or 300 μM Zn^2+^. Cells were washed twice with SBS before they were loaded with 20 μM DCFH-DA in SBS at 37 °C for 30 min, followed by extensive washing with SBS and maintaining in 200 μl of SBS. At the end of treatment with H_2_O_2_ or Zn^2+^, cells were stained by 5 μg/ml Hoechst. Images were visualized using an Olympus IX51 microscope, a digital camera and Cell^^F^ software (Olympus). The intensity of the fluorescent was quantified using ImageJ and at least 75 cells were examined in each well.

### Data presentation and statistical analysis

All data, where appropriately, are presented as mean ± standard error of mean. Statistical analysis was made using Student’s t-test for comparisons of two groups and one-way ANOVA followed by post hoc Tukey’s test for comparison among multiple groups with p < 0.05 being considered to be significant.

## Additional Information

**How to cite this article:** Mortadza, S. S. *et al*. Signalling mechanisms mediating Zn^2+^-induced TRPM2 channel activation and cell death in microglial cells. *Sci. Rep.* **7**, 45032; doi: 10.1038/srep45032 (2017).

**Publisher's note:** Springer Nature remains neutral with regard to jurisdictional claims in published maps and institutional affiliations.

## Supplementary Material

Supplementary Data

## References

[b1] Olson E. E. & McKeon R. J. Characterization of cellular and neurological damage following unilateral hypoxia/ischemia. J. Neurol. Sci. 227, 7–19 (2004). 15546586 10.1016/j.jns.2004.07.021

[b2] Wang S. W., Zhang H. & Xu Y. Crosstalk between microglia and T cells contributes to brain damage and recovery after ischemic stroke. Neurol. Res. 38, 495–503 (2016). 27244271 10.1080/01616412.2016.1188473

[b3] Szalay G. . Microglia protect against brain injury and their selective elimination dysregulates neuronal network activity after stroke. Nat. Commun. 7, 11499 (2016). 27139776 10.1038/ncomms11499 PMC4857403

[b4] Rose J. W., Hill K. E., Watt H. E. & Carlson N. G. Inflammatory cell expression of cyclooxygenase-2 in the multiple sclerosis lesion. J.Neuroimmunol. 149, 40–49 (2004). 15020063 10.1016/j.jneuroim.2003.12.021

[b5] Hill K. E., Zollinger L. V., Watt H. E., Carlson N. G. & Rose J. W. Inducible nitric oxide synthase in chronic active multiple sclerosis plaques: distribution, cellular expression and association with myelin damage. J. Neuroimmunol. 151, 171–179 (2004). 15145615 10.1016/j.jneuroim.2004.02.005

[b6] Mack C. L., Vanderlugt-Castaneda C. L., Neville K. L. & Miller S. D. Microglia are activated to become competent antigen presenting and effector cells in the inflammatory environment of the Theiler’s virus model of multiple sclerosis. J. Neuroimmunol. 144, 68–79 (2003). 14597100 10.1016/j.jneuroim.2003.08.032

[b7] Sasaki A., Yamaguchi H., Ogawa A., Sugihara S. & Nakazato Y. Microglial activation in early stages of amyloid beta protein deposition. Acta Neuropathol. 94, 316–322 (1997). 9341931 10.1007/s004010050713

[b8] Koenigsknecht J. & Landreth G. Microglial phagocytosis of fibrillar beta-amyloid through a beta(1) integrin-dependent mechanism. J.Neurosci. 24, 9838–9846 (2004). 15525768 10.1523/JNEUROSCI.2557-04.2004 PMC6730228

[b9] Gomez-Nicola D., Fransen N. L., Suzzi S. & Perry V. H. Regulation of microglial proliferation during chronic neurodegeneration. J. Neurosci. 33, 2481–2493 (2013). 23392676 10.1523/JNEUROSCI.4440-12.2013 PMC6619184

[b10] Vincenti J. E. . Defining the microglia response during the time course of chronic neurodegeneration. J. Virol. 90, 3003–3017 (2016). 10.1128/JVI.02613-15 PMC4810622 26719249

[b11] Wes P. D., Holtman I. R., Boddeke E., Moller T. & Eggen B. J. L. Next generation transcriptomics and genomics rlucidate biological complexity of microglia in health and disease. Glia 64, 197–213 (2016). 26040959 10.1002/glia.22866

[b12] Kauppinen T. M. . Zinc triggers microglial activation. J.Neurosci. 28, 5827–5835 (2008). 18509044 10.1523/JNEUROSCI.1236-08.2008 PMC2680357

[b13] Bhattacharya A. & Biber K. The microglial ATP-gated ion channel P2X7 as a CNS drug target. Glia 64, 1772–1787 (2016). 27219534 10.1002/glia.23001

[b14] Beaulieu C., Dyck R. & Cynader M. Enrichment of glutamate in zinc-containing terminals of the cat visual cortex. Neuroreport 3, 861–864 (1992). 1358251 10.1097/00001756-199210000-00010

[b15] Suh S. W. . Evidence that synaptically-released zinc contributes to neuronal injury after traumatic brain injury. Brain Res. 852, 268–273 (2000). 10678752 10.1016/s0006-8993(99)02095-8

[b16] Weiss J. H., Sensi S. L. & Koh J. Y. Zn^2+^: a novel ionic mediator of neural injury in brain disease. Trends Pharmacol. Sci. 21, 395–401 (2000). 11050320 10.1016/s0165-6147(00)01541-8

[b17] Frederickson C. J., Cuajungco M. P. & Frederickson C. J. Is zinc the link between compromises of brain perfusion (excitotoxicity) and Alzheimer’s disease? J. Alzheimers Dis. 8, 155–160 (2005). 16308484 10.3233/jad-2005-8208

[b18] Noh K. M. & Koh J. Y. Induction and activation by zinc of NADPH oxidase in cultured cortical neurons and astrocytes. J. Neurosci. 20, art. no.-RC111 (2000). 10.1523/JNEUROSCI.20-23-j0001.2000 PMC6773049 11090611

[b19] Noh K. M., Kim Y. H. & Koh J. Y. Mediation by membrane protein kinase C of zinc-induced oxidative neuronal injury in mouse cortical cultures. J.Neurochemi. 72, 1609–1616 (1999). 10.1046/j.1471-4159.1999.721609.x 10098868

[b20] Koh J. Y. Zinc and disease of the brain. Mol. Neurobiol. 24, 99–106 (2001). 11831557 10.1385/MN:24:1-3:099

[b21] Berry E. V. & Toms N. J. Pyruvate and oxaloacetate limit zinc-induced oxidative HT-22 neuronal cell injury. Neurotoxicol. 27, 1043–1051 (2006). 10.1016/j.neuro.2006.05.011 16797712

[b22] Guo D. D. . Reactive oxygen species-induced cytotoxic effects of zinc oxide nanoparticles in rat retinal ganglion cells. Toxicol. in Vitro 27, 731–738 (2013). 23232460 10.1016/j.tiv.2012.12.001

[b23] Choi D. W. & Koh J. Y. Zinc and brain injury. Annu. Rev. Neurosci. 21, 347–375 (1998). 9530500 10.1146/annurev.neuro.21.1.347

[b24] Sheline C. T., Behrens M. M. & Choi D. W. Zinc-induced cortical neuronal death: Contribution of energy failure attributable to loss of NAD^+^ and inhibition of glycolysis. J. Neurosci. 20, 3139–3146 (2000). 10777777 10.1523/JNEUROSCI.20-09-03139.2000 PMC6773145

[b25] Dineley K. E., Votyakova T. V. & Reynolds I. J. Zinc inhibition of cellular energy production: implications for mitochondria and neurodegeneration. J. Neurochem. 85, 563–570 (2003). 12694382 10.1046/j.1471-4159.2003.01678.x

[b26] Seo S. R. . Zn^2+^-induced ERK activation mediated by reactive oxygen species causes cell death in differentiated PC12 cells. J. Neurochem. 78, 600–610 (2001). 11483663 10.1046/j.1471-4159.2001.00438.x

[b27] Kim Y. H., Kim E. Y., Gwag B. J., Sohn S. & Koh J. Y. Zinc-induced cortical neuronal death with features of apoptosis and necrosis, mediation by free radicals. Neurosci. 89, 175–182 (1999). 10.1016/s0306-4522(98)00313-3 10051227

[b28] Sensi S. L., Yin H. Z., Carriedo S. G., Rao S. S. & Weiss J. H. Preferential Zn^2+^ influx through Ca^2+^-permeable AMPA/kainate channels triggers prolonged mitochondrial superoxide production. Proc. Natl. Acad. Sci. USA 96, 2414–2419 (1999). 10051656 10.1073/pnas.96.5.2414 PMC26798

[b29] Qiu L. L. . NADPH oxidase 2-derived reactive oxygen species in the hippocampus might contribute to microglial activation in postoperative cognitive dysfunction in aged mice. Brain Behav. Immun. 51, 109–118 (2016). 26254234 10.1016/j.bbi.2015.08.002

[b30] Santos C. X. C., Raza S. & Shah A. M. Redox signaling in the cardiomyocyte: From physiology to failure. Int. J. Biochem. Cell Biol. 74, 145–151 (2016). 26987585 10.1016/j.biocel.2016.03.002

[b31] Droge W. Free radicals in the physiological control of cell function. Physiol. Rev. 82, 47–95 (2002). 11773609 10.1152/physrev.00018.2001

[b32] Veal E. A., Day A. M. & Morgan B. A. Hydrogen peroxide sensing and signaling. Mol. Cell 26, 1–14 (2007). 17434122 10.1016/j.molcel.2007.03.016

[b33] Harrigan T. J., Abdullaev I. F., Jourd’heuil D. & Mongin A. A. Activation of microglia with zymosan promotes excitatory amino acid release via volume-regulated anion channels: the role of NADPH oxidases. J. Neurochem. 106, 2449–2462 (2008). 18624925 10.1111/j.1471-4159.2008.05553.x PMC2574595

[b34] Cheret C. . Neurotoxic activation of microglia is promoted by a Nox1-dependent NADPH oxidase. J. Neurosci. 28, 12039–12051 (2008). 19005069 10.1523/JNEUROSCI.3568-08.2008 PMC6671643

[b35] Deliyanti D. & Wilkinson-Berka J. L. Inhibition of NOX1/4 with GKT137831: a potential novel treatment to attenuate neuroglial cell inflammation in the retina. J. Neuroinflamm. 12, 136 (2015). 10.1186/s12974-015-0363-z PMC4518508 26219952

[b36] Walder C. E. . Ischemic stroke injury is reduced in mice lacking a functional NADPH oxidase. Stroke 28, 2252–2258 (1997). 9368573 10.1161/01.str.28.11.2252

[b37] Wu D. C. . NADPH oxidase mediates oxidative stress in the 1-methyl-4-phenyl-1,2,3,6-tetrahydropyridine model of Parkinson’s disease. Proc. Natl. Acad. Sci. USA 100, 6145–6150 (2003). 12721370 10.1073/pnas.0937239100 PMC156340

[b38] Wu D.-C., Berangere Re, D., Nagai M., Ischiropoulo H. & Przedborski S. The inflammatory NADPH oxidase enzyme modulates motor neuron degeneration in amyotrophic lateral sclerosis mice. Proc. Natl. Acad. Sci. USA 103, 12132–12137 (2006). 16877542 10.1073/pnas.0603670103 PMC1562547

[b39] Wilkinson-Berka J. L., Rana I., Armani R. & Agrotis A. Reactive oxygen species, Nox and angiotensin II in angiogenesis: implications for retinopathy. Clin. Sci. 124, 597–615 (2013). 23379642 10.1042/CS20120212

[b40] Benna J. E. . Phosphorylation of the respiratory burst oxidase subunit p67(phox) during human neutrophil activation. Regulation by protein kinase C-dependent and independent pathways. J. Biol. Chem. 272, 17204–17208 (1997). 9202043 10.1074/jbc.272.27.17204

[b41] Reeves E. P. . Direct interaction between p47(phox) and protein kinase C: evidence for targeting of protein kinase C by p47(phox) in neutrophils. Biochem. J. 344, 859–866 (1999). 10585874 PMC1220709

[b42] Min K. J. . Gangliosides activate microglia via protein kinase C and NADPH oxidase. Glia 48, 197–206 (2004). 15390122 10.1002/glia.20069

[b43] Perraud A. L. . ADP-ribose gating of the calcium-permeable LTRPC2 channel revealed by Nudix motif homology. Nature 411, 595–599 (2001). 11385575 10.1038/35079100

[b44] Sano Y. . Immunocyte Ca^2+^ influx system mediated by LTRPC2. Science 293, 1327–1330 (2001). 11509734 10.1126/science.1062473

[b45] Zou J. . A differential role of macrophage TRPM2 channels in Ca^2+^ signaling and cell death in early responses to H_2_O_2_. Am. J. Physiol.-Cell Physiol. 305, C61–C69 (2013). 23596170 10.1152/ajpcell.00390.2012

[b46] Xia R. . Identification of pore residues engaged in determining divalent cationic permeation in transient receptor potential melastatin subtype channel. J. Biol. Chem. 283, 27426–27432 (2008). 18687688 10.1074/jbc.M801049200 PMC2562080

[b47] Yamamoto S. . TRPM2-mediated Ca^2+^influx induces chemokine production in monocytes that aggravates inflammatory neutrophil infiltration. Nat. Me.d 14, 738–747 (2008). 10.1038/nm1758 PMC2789807 18542050

[b48] Zhong Z. Y.. . TRPM2 links oxidative stress to NLRP3 inflammasome activation. Nat. Commun. 4, 1611 (2013). 23511475 10.1038/ncomms2608 PMC3605705

[b49] Hecquet C. M., Ahmmed G. U., Vogel S. M. & Malik A. B. Role of TRPM2 channel in mediating H_2_O_2_-induced Ca^2+^ entry and endothelial hyperpermeability. Cir. Res. 102, 347–355 (2008). 10.1161/CIRCRESAHA.107.160176 18048770

[b50] Hecquet C. M. & Malik A. B. Role of H_2_O_2_-activated TRPM2 calcium channel in oxidant-induced endothelial injury. Thromb. Haemost. 101, 619–625 (2009). 19350103 PMC3699330

[b51] Kheradpezhouh E., Ma L. L., Morphett A., Barritt G. J. & Rychkov G. Y. TRPM2 channels mediate acetaminophen-induced liver damage. Proc. Natl. Acad. Sci. USA 111, 3176–3181 (2014). 24569808 10.1073/pnas.1322657111 PMC3939869

[b52] Gao G. F. . TRPM2 mediates ischemic kidney injury and oxidant stress through RAC1. J. Clin. Invest. 124, 4989–5001 (2014). 25295536 10.1172/JCI76042 PMC4347231

[b53] Ye M. . TRPM2 channel deficiency prevents delayed cytosolic Zn^2+^ accumulation and CA1 pyramidal neuronal death after transient global ischemia. Cell Death Dis. 5, e1541 (2014). 25429618 10.1038/cddis.2014.494 PMC4260752

[b54] Manna P. T. . TRPM2-mediated intracellular Zn^2+^ release triggers pancreatic beta-cell death. Biochem. J. 466, 537–546 (2015). 25562606 10.1042/BJ20140747

[b55] Kashio M. . Redox signal-mediated sensitization of transient receptor potential melastatin 2 (TRPM2) to temperature affects macrophage functions. Proc. Natl. Acad. Sci. USA 109, 6745–6750 (2012). 22493272 10.1073/pnas.1114193109 PMC3340098

[b56] Jiang L. H., Yang W., Zou J. & Beech D. J. TRPM2 channel properties, functions and therapeutic potentials. Expert Opin Ther. Targets 14, 973–988 (2010). 20670202 10.1517/14728222.2010.510135

[b57] Kim M. Y., Zhang T. & Kraus W. L. Poly(ADP-ribosyl)ation by PARP-1: ‘PAR-laying’ NAD^+^ into a nuclear signal. Genes Dev. 19, 1951–1967 (2005). 16140981 10.1101/gad.1331805

[b58] Chaitanya G. V., Steven A. J. & Babu P. P. PARP-1 cleavage fragments: signatures of cell-death proteases in neurodegeneration. Cell Commun. Signal. 8, 31 (2010). 21176168 10.1186/1478-811X-8-31 PMC3022541

[b59] Ying W. H., Garnier P. & Swanson R. A. NAD^+^ repletion prevents PARP-1-induced glycolytic blockade and cell death in cultured mouse astrocytes. Biochem. Biophys. Res. Commun. 308, 809–813 (2003). 12927790 10.1016/s0006-291x(03)01483-9

[b60] Yu S. W., Wang H. M., Dawson T. A. & Dawson V. L. Poly(ADP-ribose) polymerase-1 and apoptosis inducing factor in neurotoxicity. Neurobiol. Dis. 14, 303–317 (2003). 14678748 10.1016/j.nbd.2003.08.008

[b61] Kim Y. H. & Koh J. Y. The role of NADPH oxidase and neuronal nitric oxide synthase in zinc-induced poly(ADP-ribose) polymerase activation and cell death in cortical culture. Exp. Neurol. 177, 407–418 (2002). 12429187 10.1006/exnr.2002.7990

[b62] Sheline C. T., Wang H. M., Cai A. L., Dawson V. L. & Choi D. W. Involvement of poly ADP ribosyl polymerase-1 in acute but not chronic zinc toxicity. Eur. J. Neurosci. 18, 1402–1409 (2003). 14511320 10.1046/j.1460-9568.2003.02865.x

[b63] Suh S. W. . Zinc inhibits astrocyte glutamate uptake by activation of poly(ADP-ribose) polymerase-1. Mol. Med. 13, 344–349 (2007). 17728843 10.2119/2007-00043.Suh PMC1952665

[b64] Lander H. M. An essential role for free radicals and derived species in signal transduction. FASEB J. 11, 118–124 (1997). 9039953

[b65] Domercq M. . Zn^2+^-induced ERK activation mediates PARP-1-dependent ischemic-reoxygenation damage to oligodendrocytes. Glia 61, 383–393 (2013). 23281060 10.1002/glia.22441

[b66] Zhang Y. M. . Intracellular zinc release and ERK phosphorylation are required upstream of 12-lipoxygenase activation in peroxynitrite toxicity to mature rat oligodendrocytes. J. Biol. Chem. 281, 9460–9470 (2006). 16431921 10.1074/jbc.M510650200

[b67] Murray B., Alessandrini A., Cole A. J., Yee A. G. & Furshpan E. J. Inhibition of the p44/42 MAP kinase pathway protects hippocampal neurons in a cell-culture model of seizure activity. Proc. Natl. Acad. Sci. USA 95, 11975–11980 (1998). 9751775 10.1073/pnas.95.20.11975 PMC21750

[b68] Runden E. . Regional selective neuronal degeneration after protein phosphatase inhibition in hippocampal slice cultures: Evidence for a MAP kinase-dependent mechanism. J. Neurosci. 18, 7296–7305 (1998). 9736650 10.1523/JNEUROSCI.18-18-07296.1998 PMC6793243

[b69] Stanciu M. . Persistent activation of ERK contributes to glutamate-induced oxidative toxicity in a neuronal cell line and primary cortical neuron cultures. J. Biol. Chem. 275, 12200–12206 (2000). 10766856 10.1074/jbc.275.16.12200

[b70] Domercq M. . Dual-specific phosphatase-6 (Dusp6) and ERK mediate AMPA receptor-induced oligodendrocyte death. J. Biol. Chem. 286, 11825–11836 (2011). 21300799 10.1074/jbc.M110.153049 PMC3064233

[b71] Haraguchi K. . TRPM2 contributes to inflammatory and neuropathic pain through the aggravation of pronociceptive inflammatory responses in mice. J. Neurosci. 32, 3931–3941 (2012). 22423113 10.1523/JNEUROSCI.4703-11.2012 PMC6703465

[b72] Miyake T. . TRPM2 contributes to LPS/IFN gamma-induced production of nitric oxide via the p38/JNK pathway in microglia. Biochem. Biophys. Res. Commun. 444, 212–217 (2014). 24462864 10.1016/j.bbrc.2014.01.022

[b73] Kraft R. . Hydrogen peroxide and ADP-ribose induce TRPM2-mediated calcium influx and cation currents in microglia. Am. J. Physiol.-Cell Physiol 286, C129–C137 (2004). 14512294 10.1152/ajpcell.00331.2003

[b74] Fonfria E. . TRPM2 is elevated in the tMCAO stroke model, transcriptionally regulated, and functionally expressed in C13 microglia. J. Recept. Signal Transduct. Res. 26, 179–198 (2006). 16777714 10.1080/10799890600637522

[b75] Gelderblom M. . Transient receptor potential melastatin subfamily member 2 cation channel regulates detrimental immune cell invasion in ischemic stroke. Stroke 45, 3395–3402 (2014). 25236871 10.1161/STROKEAHA.114.005836

[b76] Togashi K. . TRPM2 activation by cyclic ADP-ribose at body temperature is involved in insulin secretion. EMBO J. 25, 1804–1815 (2006). 16601673 10.1038/sj.emboj.7601083 PMC1456947

[b77] Tan C. H. & McNaughton P. A. The TRPM2 ion channel is required for sensitivity to warmth. Nature 536, 460–463 (2016). 27533035 10.1038/nature19074 PMC5720344

[b78] Song K. . The TRPM2 channel is a hypothalamic heat sensor that limits fever and can drive hypothermia. Science 353, 1393–1398 (2016). 27562954 10.1126/science.aaf7537 PMC7612276

[b79] Li C. K., Meng L., Li X., Li D. L. & Jiang L. H. Non-NMDAR neuronal Ca^2+^-permeable channels in delayed neuronal death and as potential therapeutic targets for ischemic brain damage. Expert Opin. Ther. Targets 19, 879–892 (2015). 25732672 10.1517/14728222.2015.1021781

[b80] Mortadza S. A. S., Wang L., Li D. L. & Jiang L. H. TRPM2 channel-mediated ROS-sensitive Ca^2+^ signaling mechanisms in immune cells. Front. Immunol. 6, 407 (2015). 26300888 10.3389/fimmu.2015.00407 PMC4528159

[b81] Takahashi N., Kozai D., Kobayashi R., Ebert M. & Mori Y. Roles of TRPM2 in oxidative stress. Cell Cal. 50, 279–287 (2011). 10.1016/j.ceca.2011.04.006 21616534

[b82] Combs C. K., Johnson D. E., Cannady S. B., Lehman T. M. & Landreth G. E. Identification of microglial signal transduction pathways mediating a neurotoxic response to amyloidogenic fragments of beta-amyloid and prion proteins. J. Neurosci. 19, 928–939 (1999). 9920656 10.1523/JNEUROSCI.19-03-00928.1999 PMC6782151

[b83] Rolon-Reyes K. . Microglia activate migration of glioma cells through a Pyk2 intracellular pathway. Plos One 10, e0131059 (2015). 26098895 10.1371/journal.pone.0131059 PMC4476590

[b84] Lev S. . Protein tyrosine kinase PYK2 involved in Ca^2+^-induced regulation of ion channel and MAP kinase functions. Nature 376, 737–745 (1995). 7544443 10.1038/376737a0

[b85] Yao H. . TRPC channel-mediated neuroprotection by PDGF involves Pyk2/ERK/CREB pathway. Cell Death Differ. 16, 1681–1693 (2009). 19680266 10.1038/cdd.2009.108 PMC2783976

[b86] Hara H., Taniguchi M., Kobayashi M., Kamiya T. & Adachi T. Plasma-activated medium-induced intracellular zinc liberation causes death of SH-SY5Y cells. Arch. Biochem. Biophys. 584, 51–60 (2015). 26319292 10.1016/j.abb.2015.08.014

[b87] Sloviter R. S. A selective loss of hippocampal mossy fiber Timm stain accompanies granule cell seizure activity induced by perforant path stimulation. Brain Res. 330, 150–153 (1985). 2859083 10.1016/0006-8993(85)90017-4

[b88] Koh J. Y. . The role of zinc in selective neuronal death after transient global cerebral ischemia. Science 272, 1013–1016 (1996). 8638123 10.1126/science.272.5264.1013

[b89] Ostapchenko V. G. . The transient receptor potential melastatin 2 (TRPM2) channel contributes to beta-amyloid oligomer-related neurotoxicity and memory impairment. J. Neurosci. 35, 15157–15169 (2015). 26558786 10.1523/JNEUROSCI.4081-14.2015 PMC6605355

[b90] Wu L. J. . The voltage-gated proton channel Hv1 enhances brain damage from ischemic stroke. Nat. Neurosci. 15, 565–573 (2012). 22388960 10.1038/nn.3059 PMC3314139

[b91] Yang W. . Zinc inactivates melastatin transient receptor potential 2 Channels via the outer pore. J. Biol. Chem. 286, 23789–23798 (2011). 21602277 10.1074/jbc.M111.247478 PMC3129160

