# Supplementary material for: Signalling mechanisms mediating Zn2+-induced TRPM2 channel activation and cell death in microglial cells
Source: Sci Rep. 2017 Mar 21;7:45032. doi: 10.1038/srep45032 (PMC5359577; doi:10.1038/srep45032)

Supplemental data

Signalling mechanisms mediating Zn^{2+}-induced TRPM2 channel activation and death cell in microglial cells

Sharifah Syed Mortadza[1], Joan A Sim[2], Martin Stacey[3] and Lin-Hua Jiang[1,4*]

[1]School of Biomedical Sciences, Faculty of Biological Sciences, University of Leeds, United Kingdom; [2]School of Life Sciences, University of Manchester, United Kingdom; and [3]School of Molecular and Cell Biology, Faculty of Biological Sciences, University of Leeds, United Kingdom; [4]Sino-UK Joint Laboratory of Brain Function and Injury, and Department of Physiology and Neurobiology, Xinxiang Medical University, PR China

*Correspondence: Dr Lin-Hua Jiang, School of Biomedical Sciences, Faculty of Biological Sciences, University of Leeds, Leeds LS2 9JT, United Kingdom. Email: l.h.jiang@leeds.ac.uk; Telephone: (+)44 (0)113 3434231

Running title: TRPM2 in Zn^{2+}-induced microglial cell death

*Correspondence: Dr Lin-Hua Jiang, School of Biomedical Sciences, Faculty of Biological Sciences, University of Leeds, Leeds LS2 9JT, United Kingdom. Email: l.h.jiang@leeds.ac.uk; Telephone: (+)44 (0)113 3434231

Fig. 1 H_2O_2-induced increase in the $[Ca^{2+}]_c$ in microglial cells is inhibited by PJ34 and temperature-dependent.

(**a**) *Left*, representative single cell images showing Ca^{2+} responses (top row: Fluo-4 fluorescence; bottom row: co-staining with Hoechst) of microglial cells to 300 μM H_2O_2 without and with treatment with 10 μM PJ34. Cells were treated with PJ34 for 30 min prior to and during exposure to H_2O_2. *Right*, summary of mean H_2O_2-induced Ca^{2+} responses in microglial cells under indicated conditions from three independent experiments, using three wells of cells for each condition in each experiment. (**b**) Representative single cell images showing Ca^{2+} responses (top row: Fluo-4 fluorescence; bottom row: co-staining with Hoechst) of microglial cells to 100-300 μM H_2O_2 at 37°C and 22°C. (c) Summary of H_2O_2-induced Ca^{2+} responses at 37°C and 22°C, from 3 independent experiments, using three wells of cells for each condition in each experiment.. Scale bar, 40 μm. **, $p < 0.01$ and ***, $p < 0.005$ compared to the respective control group. ###, $p < 0.005$ compared between cells under the same treatment but at different temperatures.

Fig. 2 H_2O_2 induces microglial cell death via necrosis

(**a-c**) *Left*, representative images showing microglial cell death (top row: PI-stained dead cells; bottom row: co-staining with Hoechst) after cells were exposed for 24 hrs to 300 μM H_2O_2 alone or together with 1 μM IM-54 (a) or 30 μM Ac-DEVD-CMK (aDC) (b) or BAPTA-AM at indicated concentrations (c). Cells were treated with the inhibitors for 30 min prior to and during exposure to H_2O_2. Scale bar, 20 μm. *Right*, summary of the mean percentage of cell death induced by 300 μM H_2O_2 under indicated conditions from three independent experiments, using three wells of cells for each condition in each experiment. **, $p < 0.05$; ***, $p < 0.005$ compared to indicated control group. Treatment with aDC resulted in no significant effect on H_2O_2-induced cell death.

Fig. 3 Zn^{2+} induces microglial cell death via necrosis

(**a, b**) *Left*, representative images showing microglial cell death (top row: PI-stained dead cells; bottom row: co-staining with Hoechst) after cells were exposed for 24 hrs to 300 μM Zn^{2+} together with IM-54 (a) or Ac-DEVD-CMK (aDC) (b) at indicated concentrations. Cells were treated with the inhibitors for 30 min prior to and during exposure to Zn^{2+}. Scale bar, 20 μm. *Right*, summary of the mean percentage of cell death from three independent experiments, using three wells of cells for each condition in each experiment. ***, $p < 0.005$ compared to indicated control group. Treatment with aDC resulted in no significant effect on Zn^{2+}-induced cell death.

Fig. 4 Inhibition of Zn^{2+}-induced microglial cell death by TRPM2 channel inhibitors

Representative images showing microglial cell death (top row: PI-stained dead cells; bottom row: co-staining with Hoechst) after cells were exposed for 24 hrs to 300 μM Zn^{2+} together with 10 μM PJ34 (a), 10 μM DPQ (b) or 100 μM 2-APB (c). Cells were treated with the inhibitors for 30 min prior to and during exposure to Zn^{2+}.

Fig. 5 Zn^{2+}-induced Ca^{2+} responses in microglial cells are temperature-dependent and inhibited by PJ34.

(**a**) Representative single cell images showing Ca^{2+} responses (top row: Fluo-4 fluorescence; bottom row: co-staining with Hoechst) of microglial cells to 100-300 μM Zn^{2+} at 37°C and 22°C. (b) Summary of the mean Ca^{2+} responses at 37°C and 22°C, from 3 independent experiments. (c) Representative single cell images (left panel, top row: Fluo-4 fluorescence; bottom row: co-staining with Hoechst) and mean data (right panel) showing Ca^{2+} responses of microglial cells to 300 μM Zn^{2+} without and with treatment with 10 μM PJ-34. Cells were treated with the inhibitors for 30 min prior to and during exposure to Zn^{2+}.

Fig. 6 Involvement of NADPH oxidase activation in Zn^{2+}-induced cell death, ROS generation, PARP-1 activation, increase in the $[Ca^{2+}]_c$ in microglial cells

(**a**) Representative images showing cell death (top row: PI-stained dead cells; bottom row: all cells stained with Hoechst) in microglial cells exposed for 24 hrs to 300 μM Zn^{2+} alone or together with 3 μM GKT137831 (GKT). (**b**) *R*epresentative images showing DCF fluorescence (top row) and Hoechst (middle row) and merged images (bottom row) in cells exposed for 2 hrs to 300 μM Zn^{2+} alone or together with 1 μM GKT. (**c**) Representative images showing PAR staining (top row) and DAPI (middle row) and merged images (bottom row) of cells exposed for 2 hrs to 300 μM Zn^{2+} alone or together with 3 μM GKT. (**d**) Representative single cell images showing Ca^{2+} responses in microglial cells (top row: Fluo-4 fluorescence; bottom row: count-staining with Hoechst) exposed for 2 hrs to 300 μM Zn^{2+} alone or together with for 2 hrs to 300 μM Zn^{2+} alone or together with 1 μM GKT). Cells were treated with DPI or GKT for 30 min prior to and during exposure to Zn^{2+}. Scale bar, 20 μm (a) and 40 μm (b-d).

Fig. 7 Effects of NOX2 inhibitor on in Zn^{2+}-induced cell death, ROS generation, PARP-1 activation, increase in the $[Ca^{2+}]_c$ in microglial cells

(**a**) *Left* representative images showing cell death (top row: PI-stained dead cells; bottom row: co-staining with Hoechst) in microglial cells exposed for 24 hrs to 300 μM Zn^{2+} alone or together with 30 μM Phox-I2 (Phox). (**b**) *Left*, representative images showing DCF fluorescence (top row) and co-staining with Hoechst (bottom row) in cells exposed for 2 hrs to 300 μM Zn^{2+} alone or together with 30 μM Phox. (**c**), *Left* representative images showing PAR staining (top row) and co-staining with DAPI (bottom row) of cells exposed for 2 hrs to 300 μM Zn^{2+} alone or together with 30 μM Phox. (**d**) *Left*, representative single cell images showing Ca^{2+} responses in microglial cells (top row: Fluo-4 fluorescence; bottom row: count-staining with Hoechst) exposed for 2 hrs to 300 μM Zn^{2+} alone or together with for 2 hrs to

300 μM Zn^{2+} alone or together with 30 μM Phox. Cells were treated with Phox for 30 min prior to and during exposure to Zn^{2+}. Scale bar, 20 μm (a) and 40 μm (all other panels). (**a-d**) *Right,* summary of the mean data from three independent experiments, using three wells of cells for each condition in each experiment. Scale bar, 20 μm (a) and 40 μm (b-d). *, $p < 0.05$; ***, $p < 0.005$ compared to the indicated control group exposed to with Zn^{2+} alone. Treatment with the highest concentration of Phox (a) alone resulted in no significant cell death.

Fig. 8 The PKC/NOX signalling pathway is required for, and the PYK2/MEK signalling pathway depends on, the TRPM2 channel activation

Left, representative images showing the Ca^{2+} responses (top row: Fluo-4 fluorescence; bottom row: count-staining with Hoechst) in the WT microglia cells, treated with 1 μM PF 431396 (PF) (a) or 10 μM U0126 (b) alone or together with 1 μM CTC, 3 μM DPI, or 1 μM GKT137831 (GKT) 30 min prior and during 2 hrs exposure to 300 μM Zn^{2+}. *Right*, summary of the mean Ca^{2+} responses in cells under indicated conditions from at least three independent experiments, using three wells of cells for each condition in each experiment. In the presence of PF to inhibit PYK2 (a) or U0126 to inhibit MER/ERK (b), Zn^{2+} induced significant increase in the $[Ca^{2+}]_c$ in the WT microglial cells, which was strongly inhibited by treatment with CTC, DPI or GKT. Scale bar, 40 μm. ***, $p < 0.005$ compared to indicated control group treated with PF or U0126 alone, and ###, $p < 0.005$ compared to cells exposed to Zn^{2+} and treated with PF or U0126.

Fig. 9 PKC is not involved in H_2O_2-induced PARP-1 activation and microglial cell death

(**a**) *Left*, representative images showing PAR staining (top row) and co-staining with DAPI (bottom row) of microglial cells exposed for 2 hrs to 300 μM H_2O_2 alone or together with chelerythrine chloride (CTC) at indicated concentrations. Cells were treated with CTC for 30

min prior to and during exposure to H_2O_2. *Right*, summary of the mean PAR fluorescence intensity in cells under indicated conditions from three independent experiments, using three wells of cells for each condition in each experiment. (**b**) *Left*, representative images showing microglial cell death (top row: PI-stained dead cells; bottom row: all cells stained with Hoechst) after cells were exposed for 24 hrs to 300 μM H_2O_2 for 24 hrs. Cells were treated with CTC for 30 min prior to and during exposure to H_2O_2. *Right*, summary of the mean percentage of cell death from three independent experiments, using three wells of cells for each condition in each experiment. Scale bar, 40 μm (a) and 20 μm (b). Treatment with CTC resulted in no significant effect on H_2O_2-induced increase in PAR staining and cell death.

Fig. 10 NOX is not involved in H_2O_2-induced PARP-1 activation and microglial cell death (**a**, **c**) *Left*, representative images showing PAR (top row) and co-staining DAPI staining (bottom row) of cells exposed for 2 hrs to 300 μM H_2O_2 alone or together with 3 μM DPI (a) or 3 μM GKT137831 (GKT) (c). *Right*, summary of the mean PAR fluorescence intensity in cells under indicated conditions from three independent experiments, using three wells of cells for each condition in each experiment. (**b, d**) *Left*, representative images showing microglial cell death (top row: PI-stained dead cells; bottom row: co-staining with Hoechst) after cells were exposed for 24 hrs to 300 μM H_2O_2 alone or together with 3 μM DPI (b) or 3 μM GKT (d). *Right*, summary of the mean percentage of cell death under indicated conditions from three independent experiments, using three wells of cells for each condition in each experiment. Cells were treated with DPI or GKT for 30 min prior to and during exposure to H_2O_2. Scale bar, 40 μm (a, c) and 20 μm (b, d). Neither DPI nor GKT treatment resulted in significant effect on H_2O_2-induced increase in PAR staining and cell death.

Fig. 11 The PYK2/MEK signalling pathway is not involved in H_2O_2-induced PARP-1 activation and microglial cell death

(**a, c**) *Left*, representative images showing PAR (top row) and co-staining with DAPI staining (bottom row) of cells exposed for 2 hrs to 300 μM H_2O_2 alone or together with 1 μM PF431396 (PF) (a) or 10 μM U0126 (c). *Right*, summary of the mean PAR fluorescence intensity in cells under the indicated conditions, from three independent experiments, using three wells of cells for each condition in each experiment. (**b, d**) *Left*, representative images showing microglial cell dead (top row: PI-stained dead cells; bottom row: co-staining with Hoechst) after cells were exposed for 24 hrs to 300 μM H_2O_2 alone or together with 1 μM PF (b) or 10 μM U0126 (d). *Right*, summary of the mean percentage of cell death under indicated conditions from three independent experiments, using three wells of cells for each condition in each experiment. Cells were treated with PF or U0126 for 30 min prior to and during exposure to H_2O_2. Scale bar, 40 μm (a, c) and 20 μm (c, d). Neither PF nor U0126 treatment resulted in significant effect on H_2O_2-induced increase in PAR staining and cell death.

Supplemental Fig. 1

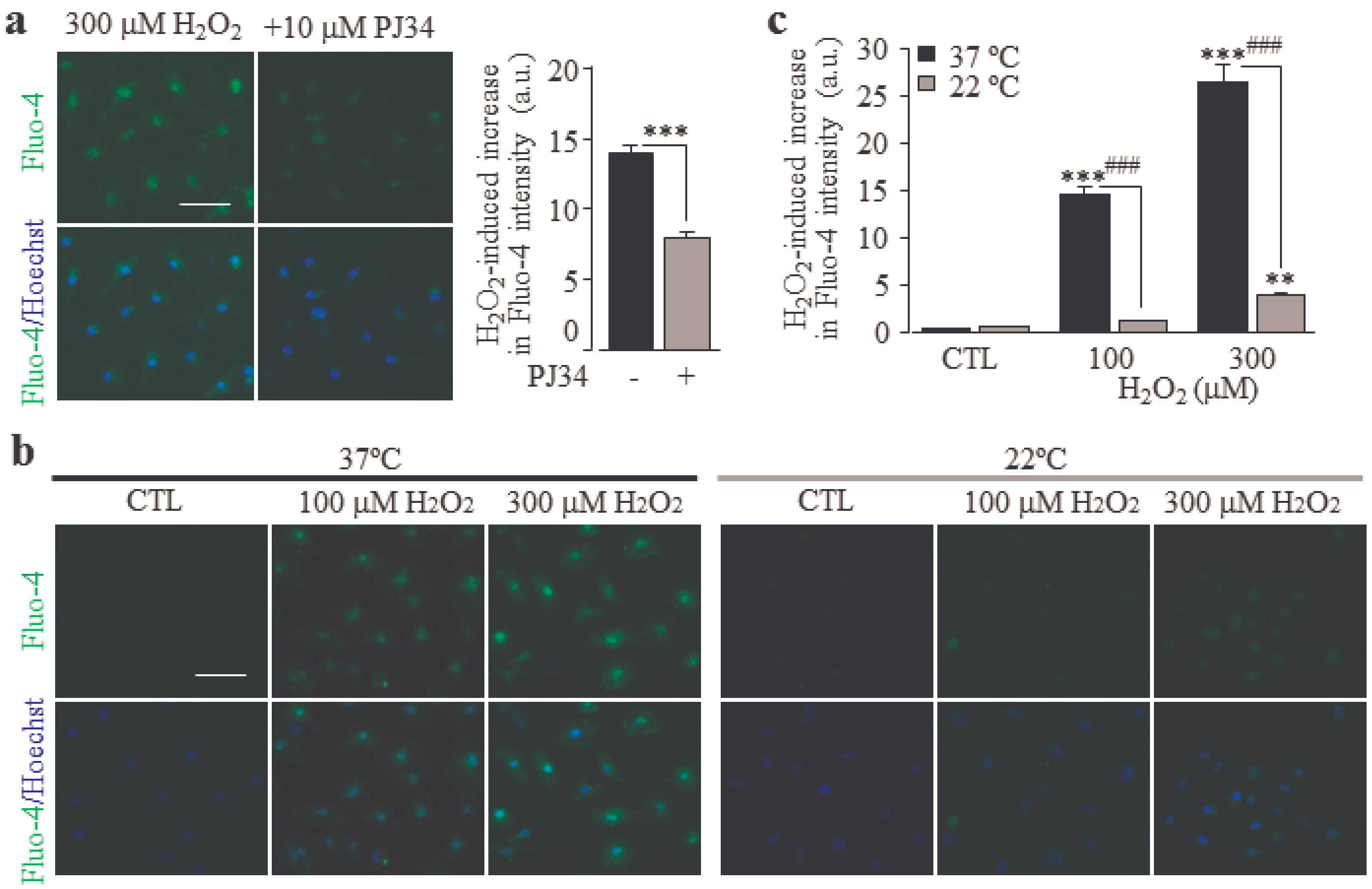

Supplemental Fig. 2

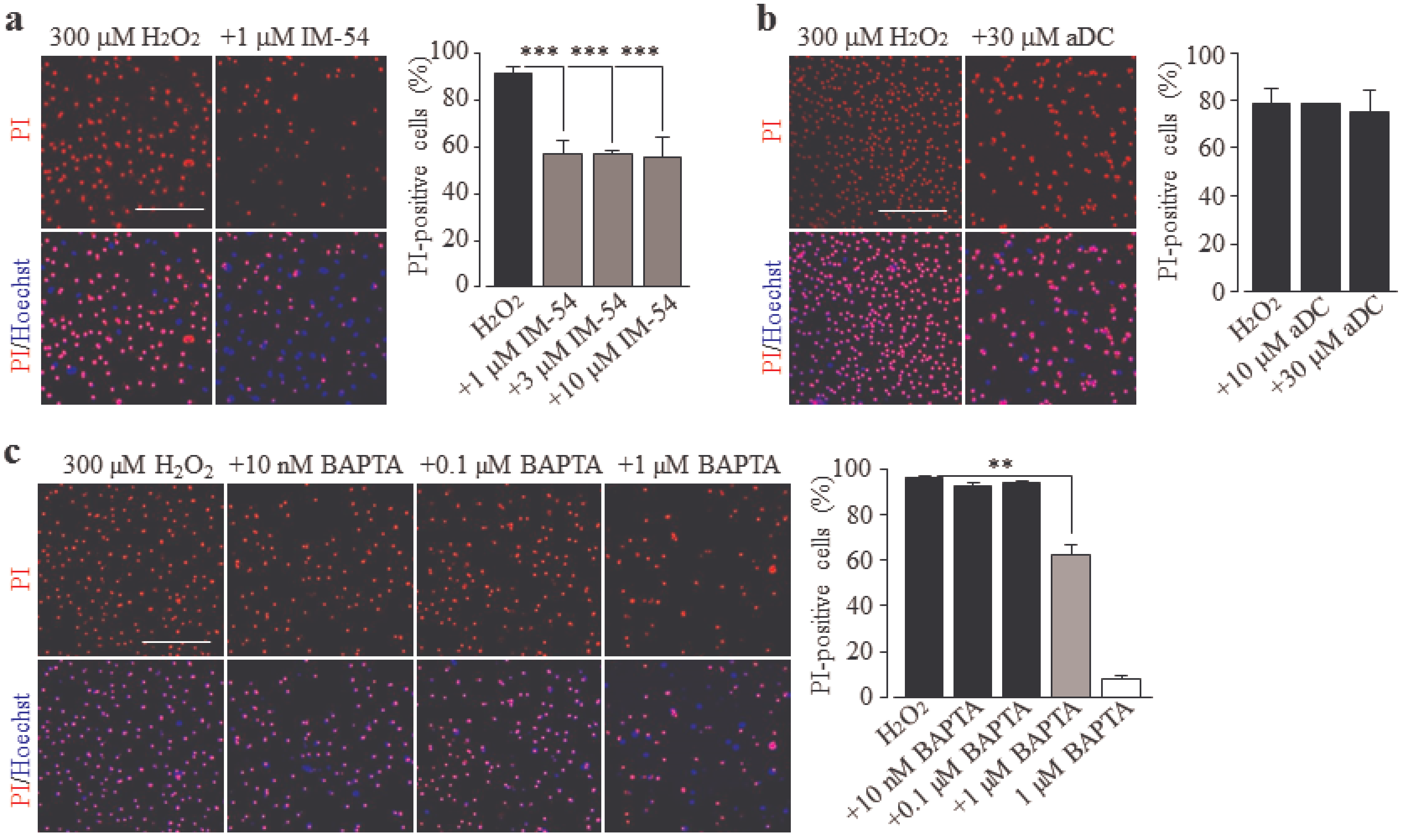

Supplemental Fig. 3

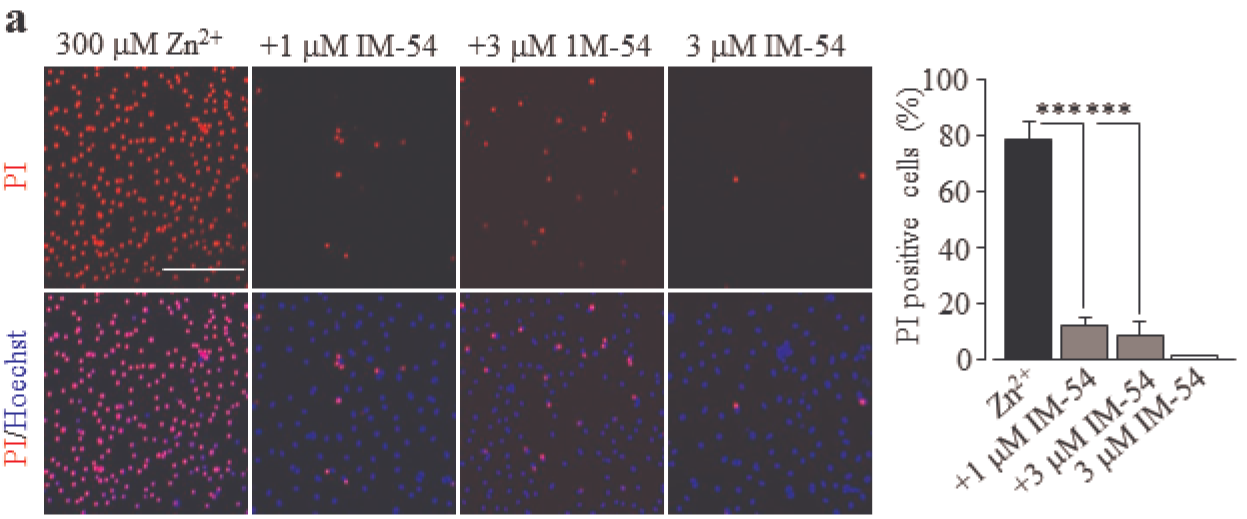

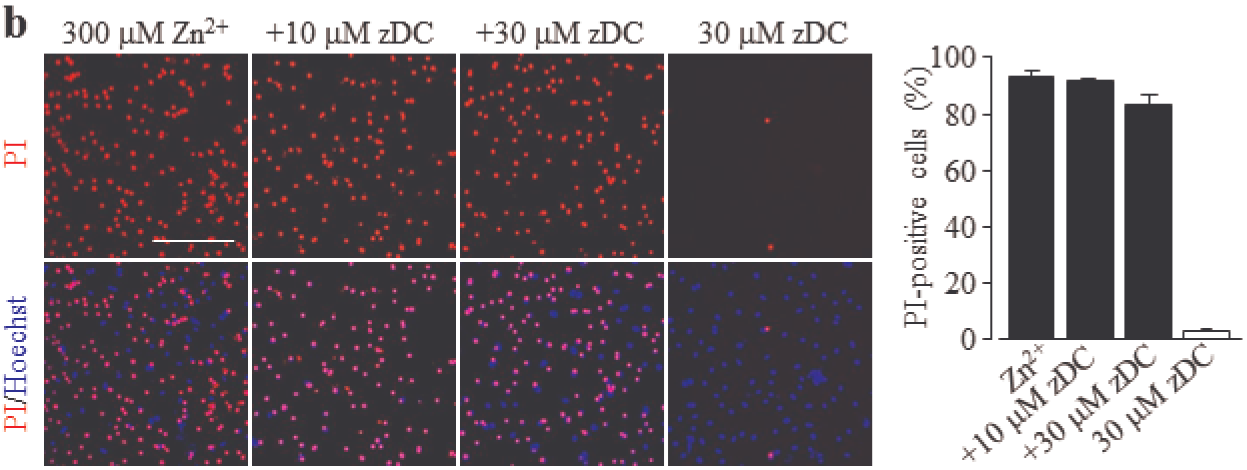

Supplemental Fig. 4

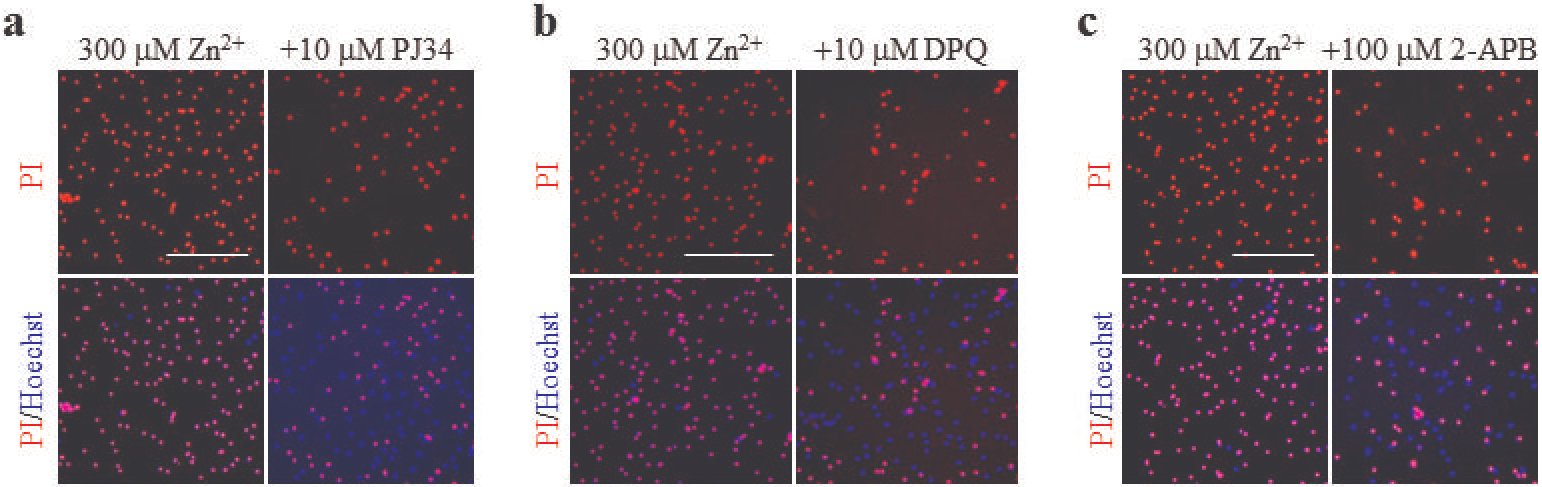

Supplemental Fig. 5

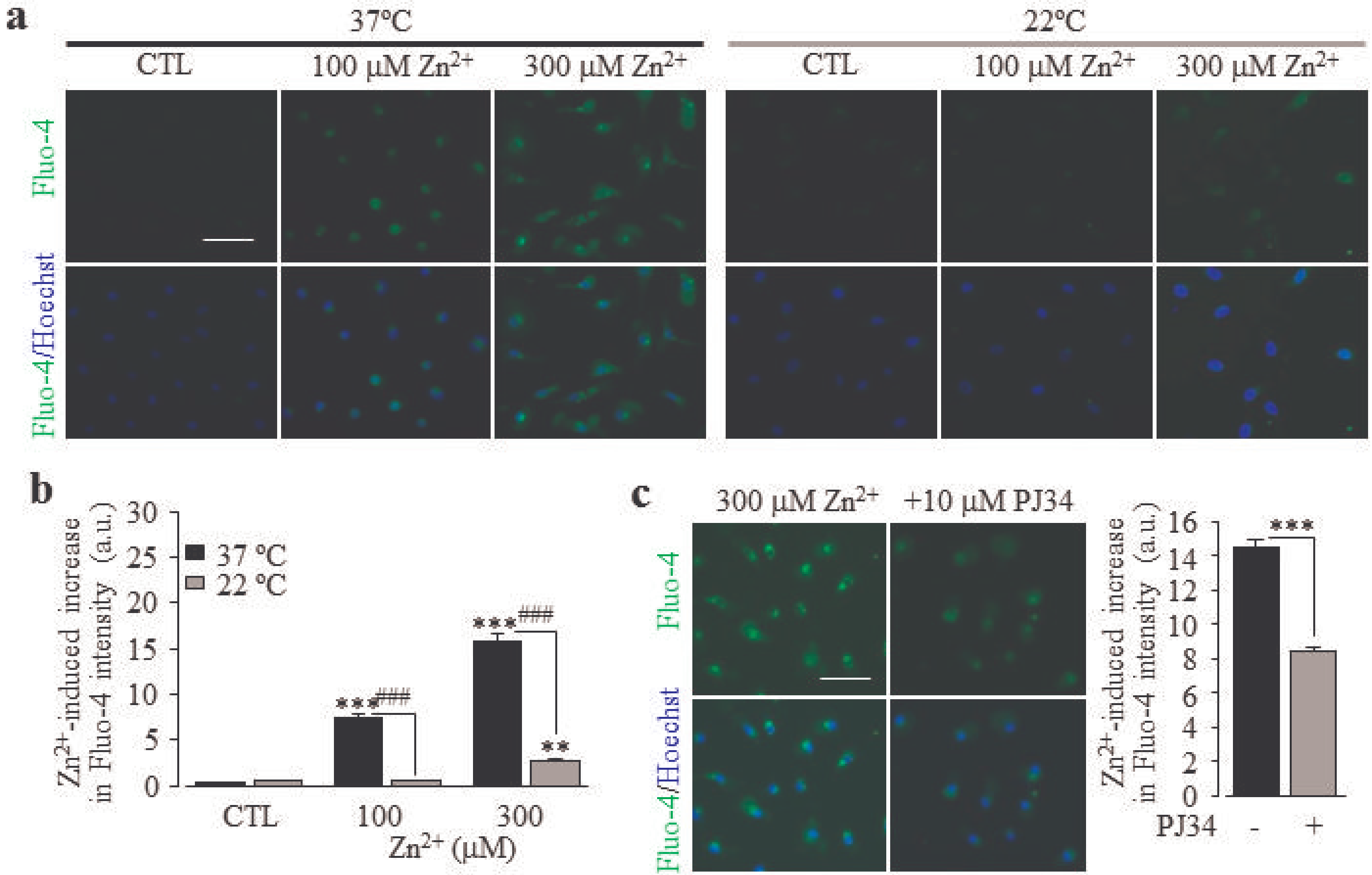

Supplemental Fig. 6

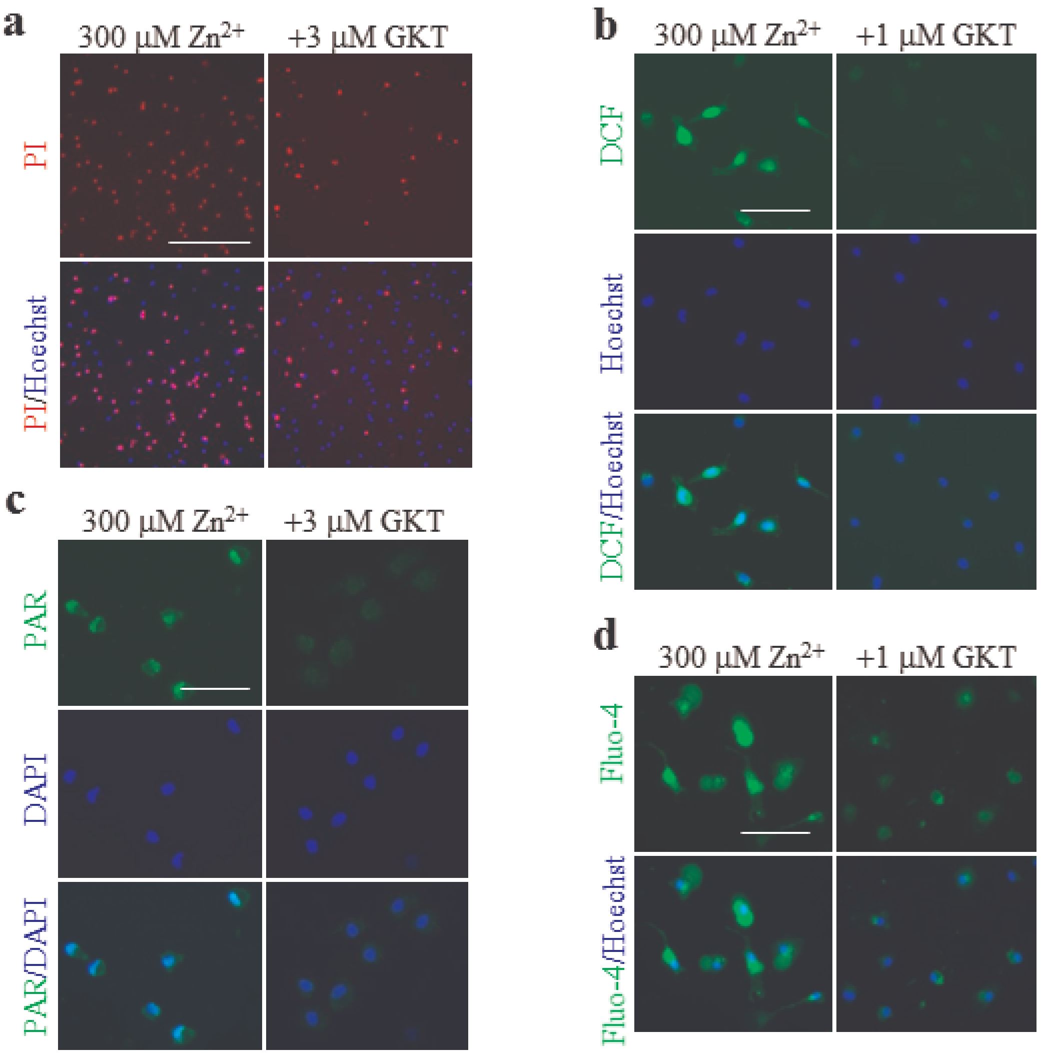

Supplemental Fig. 7

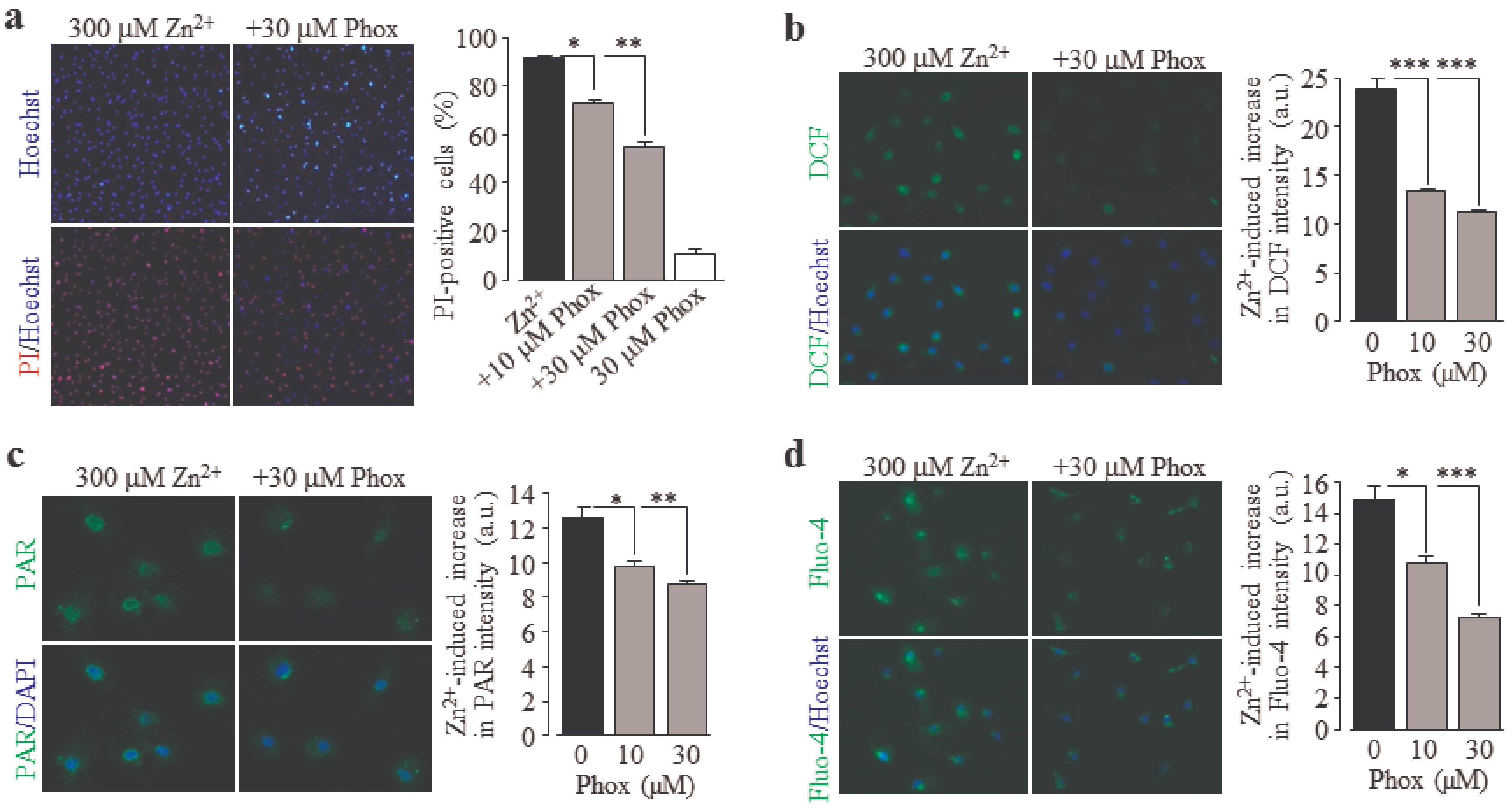

Supplemental Fig. 8

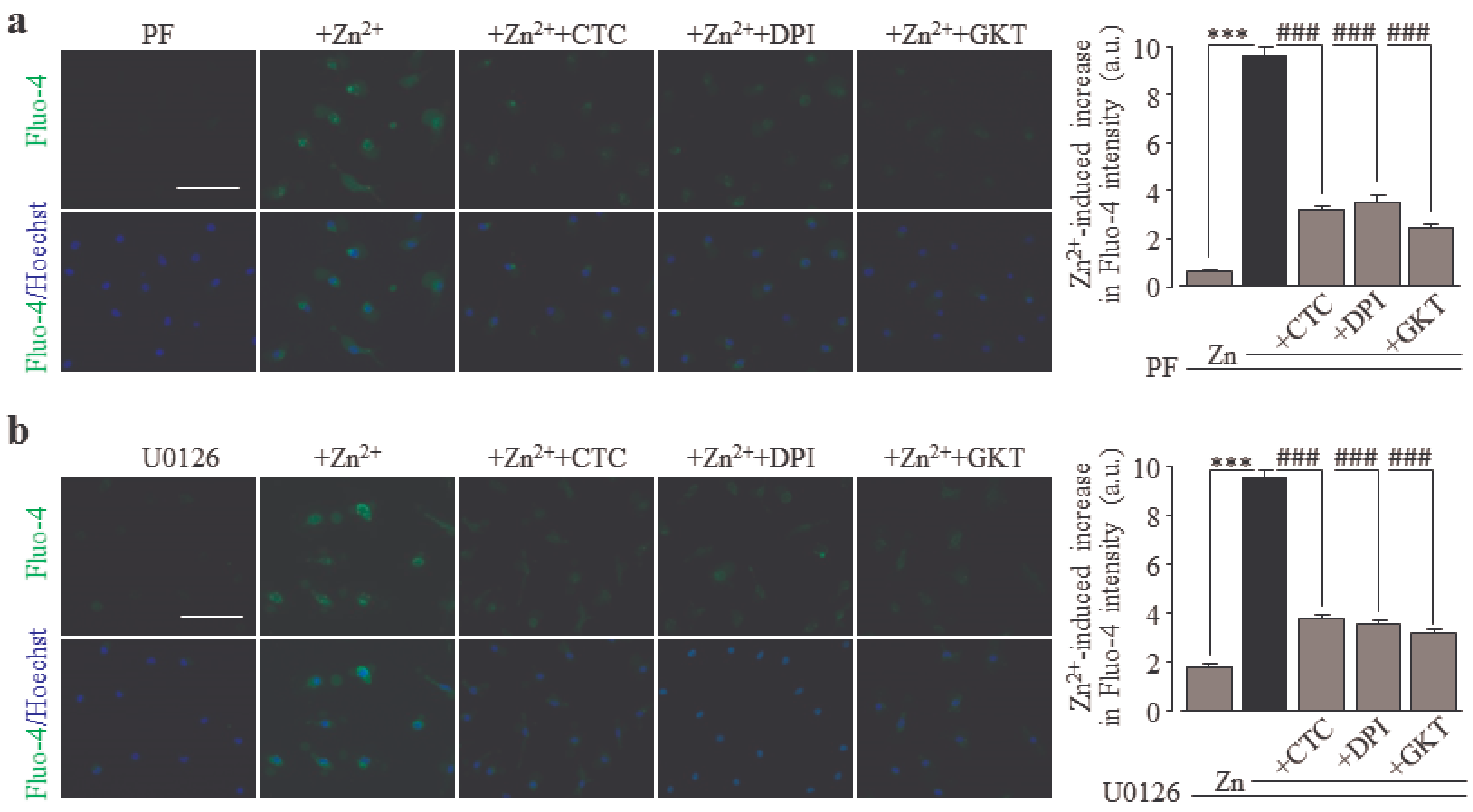

Supplemental Fig. 9

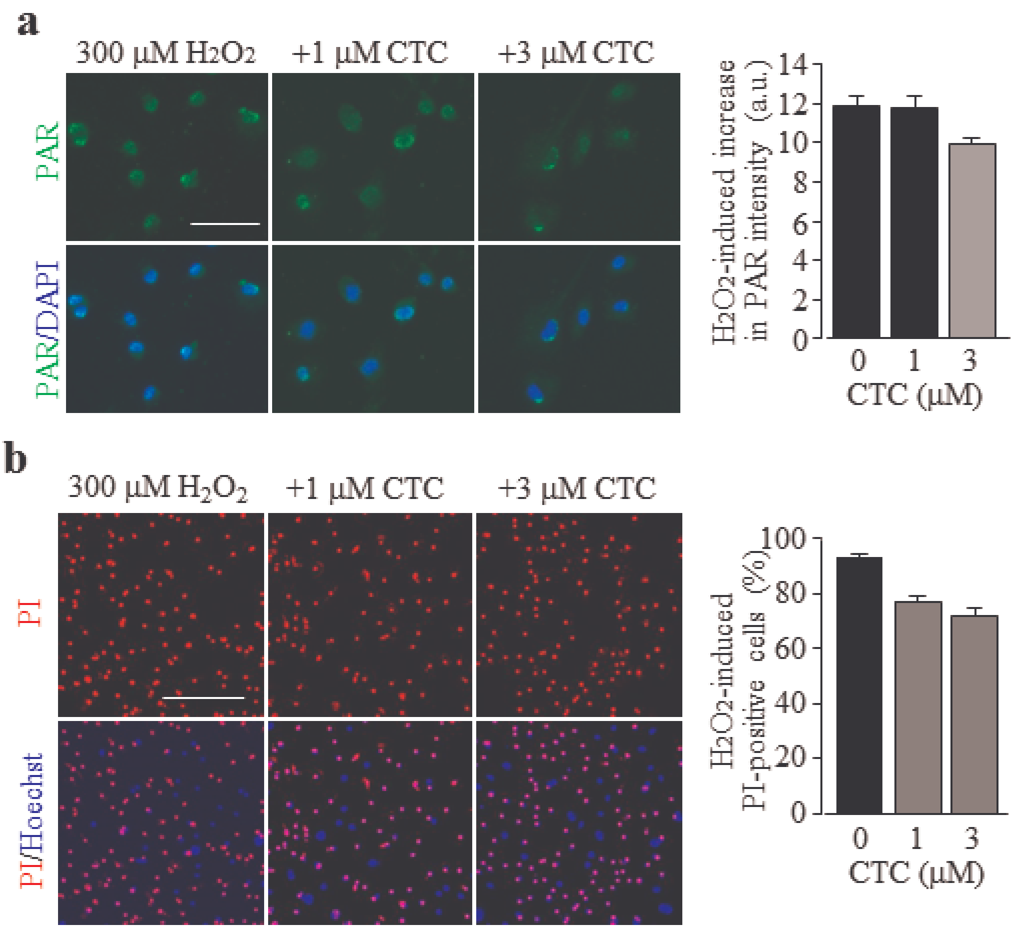

Supplemental Fig. 10

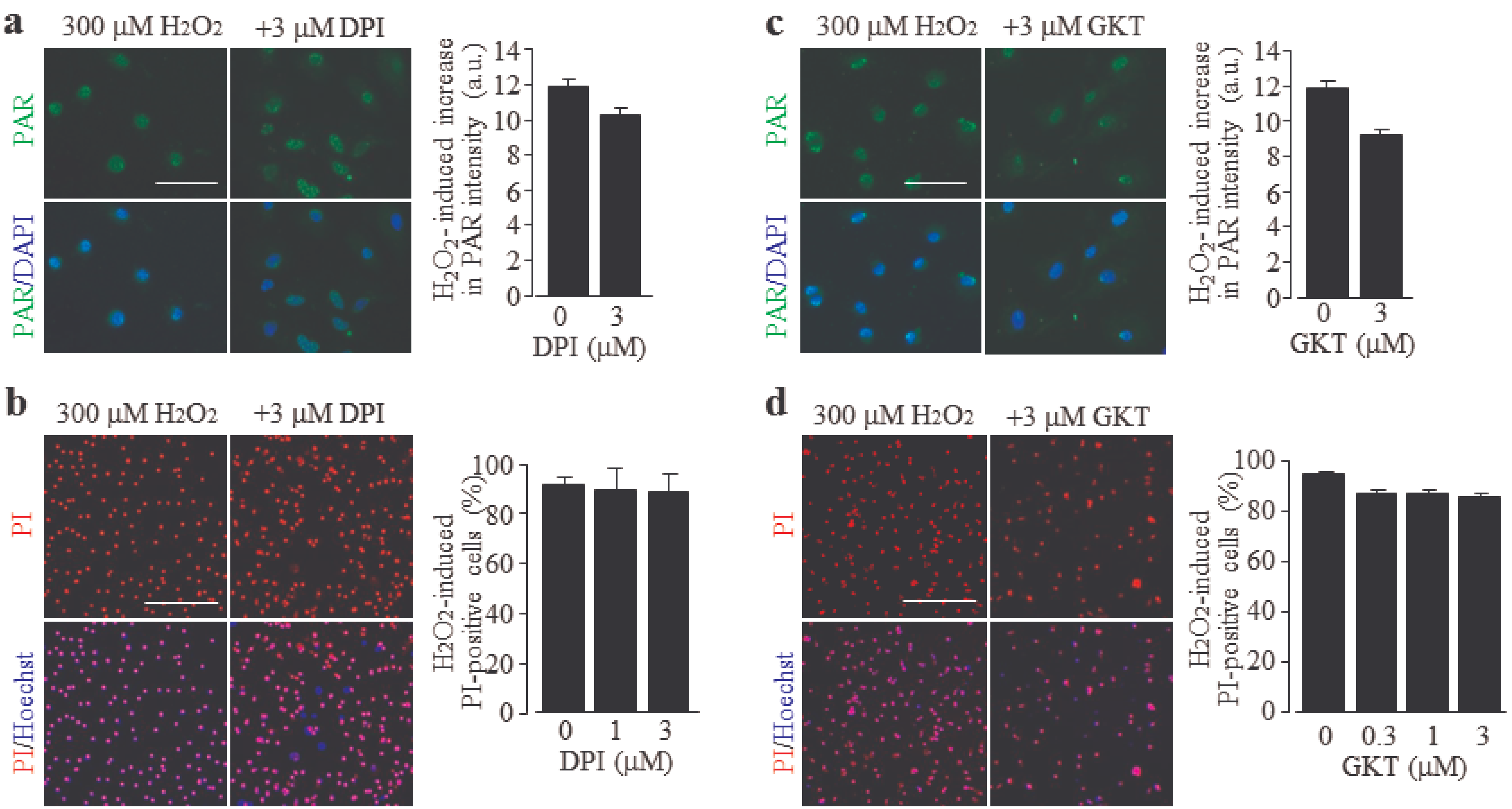

Supplemental Fig. 11

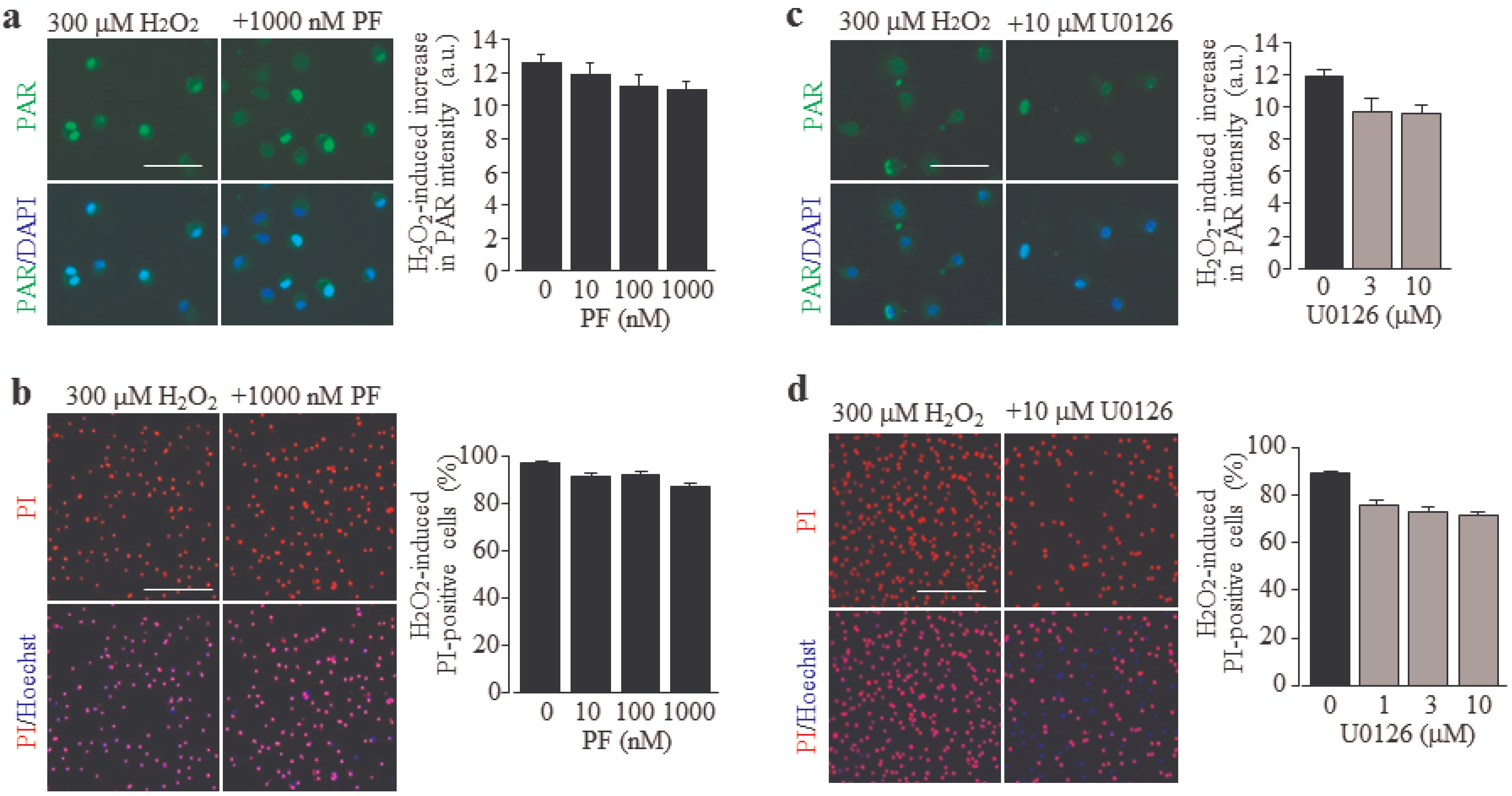

Supplement: Supplementary Data [file srep45032-s1.pdf]
